# A new species of *Leptobrachella* Smith 1925 (Anura, Megophryidae) from Lai Chau Province, Vietnam

**DOI:** 10.3897/BDJ.12.e136491

**Published:** 2024-11-04

**Authors:** Chung Van Hoang, Anh Mai Luong, Truong Quang Nguyen, Tao Thien Nguyen, Hoa Thi Ninh, Linh Hoang Tu Le, Thomas Ziegler, Cuong The Pham

**Affiliations:** 1 Institute of Ecology and Biological Resources, Vietnam Academy of Science and Technology, Hanoi, Vietnam Institute of Ecology and Biological Resources, Vietnam Academy of Science and Technology Hanoi Vietnam; 2 Forest Resources and Environment Center, Forest Inventory and Planning Institute, Hanoi, Vietnam Forest Resources and Environment Center, Forest Inventory and Planning Institute Hanoi Vietnam; 3 Graduate University of Science and Technology, Vietnam Academy of Science and Technology, Hanoi, Vietnam Graduate University of Science and Technology, Vietnam Academy of Science and Technology Hanoi Vietnam; 4 Institute of Genome Research, Vietnam Academy of Science and Technology, Hanoi, Vietnam Institute of Genome Research, Vietnam Academy of Science and Technology Hanoi Vietnam; 5 Zoological Garden, Cologne, Germany Zoological Garden Cologne Germany; 6 Institute of Zoology, University of Cologne, Cologne, Germany Institute of Zoology, University of Cologne Cologne Germany

**Keywords:** *Leptobrachellahuynhi* sp. nov., Sin Ho, Hoang Lien Range, molecular phylogeny, taxonomy

## Abstract

**Background:**

The genus *Leptobrachella* (Anura, Megophryidae) was originally described, based on the type species from Sarawak (Malaysia), *Leptobrachella mjöbergi* Smith. The taxa in the group were previously classified into different genera, i.e, *Paramegophrys* Liu; *Leptolalax* Dubois; *Lalax* Delorme, Dubois, Grosjean & Ohler; and *Lalos* Dubois, Grosjean, Ohler, Adler & Zhao. However, Yuan et al. synonymised *Leptolalax* with *Leptobrachella* in 2017. Members of *Leptobrachella* inhabit the forest floor and rocky streams in hilly evergreen forests. They are widely distributed from southern China and Myanmar through mainland Indochina to Peninsular Malaysia and the island of Borneo. However, the species diversity of the genus was indicated to be underestimated by phylogenetic analyses and a series of new species have been discovered recently. In Vietnam, 34 species of *Leptobrachella* are currently known and 75% (or 24 species) have been described or newly recorded from the country since 2010.

**New information:**

We describe a new species, *Leptobrachellahuynhi* sp. nov., from Sin Ho District, Lai Chau Province. The new species is distinguished from its congeners by genetic divergences ranging from 3.62 to 18.51% (16S rRNA gene) and morphological differences: size medium (SVL 37.8–40.2 mm in adult females); head longer than wide; tympanum distinct; skin on entire dorsum shagreened; toes without webbing and with narrow lateral fringes; supratympanic ridge slightly rough with few nodules; dorsum grey-brown with indistinct dark brown markings; an interorbital region with a stacking double Y-shaped marking; centre of belly creamy-white, outer edges of belly brown with small whitish spots; iris copper. The new species is the 35^th^ species of the genus *Leptobrachella* known from Vietnam.

## Introduction

Due to its geographic position in the transition zone between cold climate in the Tibetan mountains of China and the subtropical mountains of Southeast Asia, the northwest of Vietnam is likely to harbour particularly high level of biodiversity and may contain previously unknown herpetofaunal taxa (Sterling et al. 2006). A series of new amphibians have been described from the Hoang Lien Range in north-western Vietnam during the last decade, including six species from Lao Cai Province (*Leptobrachellabotsfordi* (Rowley, Dau & Nguyen, 2013); *Oreolalaxsterlingae* Nguyen, Phung, Le, Ziegler & Böhme, 2013; *Boulenophrysrubrimera* (Tapley, Cutajar, Mahony, Chung, Dau, Nguyen, Luong & Rowley, 2017); *Gracixalussapaensis* Matsui, Ohler, Eto & Nguyen, 2017; *Boulenophrysfansipanensis* (Tapley, Cutajar, Mahony, Nguyen, Dau, Luong, Le, Nguyen, Nguyen, Portway, Luong & Rowley, 2018); *B.hoanglienensis* (Tapley, Cutajar, Mahony, Nguyen, Dau, Luong, Le, Nguyen, Nguyen, Portway, Luong & Rowley, 2018) and four species from Lai Chau Province (*B.frigida* (Tapley, Cutajar, Nguyen, Portway, Mahony, Nguyen, Harding, Luong & Rowley, 2021); *Leptobrachellagraminicola* Nguyen, Tapley, Nguyen, Luong & Rowley, 2021); *Tylototritonsparreboomi* Bernardes, Le, Nguyen, Pham, Pham, Nguyen & Ziegler, 2020; and *Microhylahmongorum* Hoang, Nguyen, Phan, Pham, Ninh, Wang, Jiang, Ziegler & Nguyen, 2022) (Frost 2024).

During our fieldwork in the evergreen forests of the Hoang Lien Range in north-western Vietnam, four specimens of *Leptobrachella* were collected in Sin Ho District of Lai Chau Province, Vietnam. Detailed morphological and molecular analyses revealed that these specimens represent a distinct unnamed taxon which we herein describe as a new species of *Leptobrachella*.

## Materials and methods

**Sampling**

**Figure 1.**
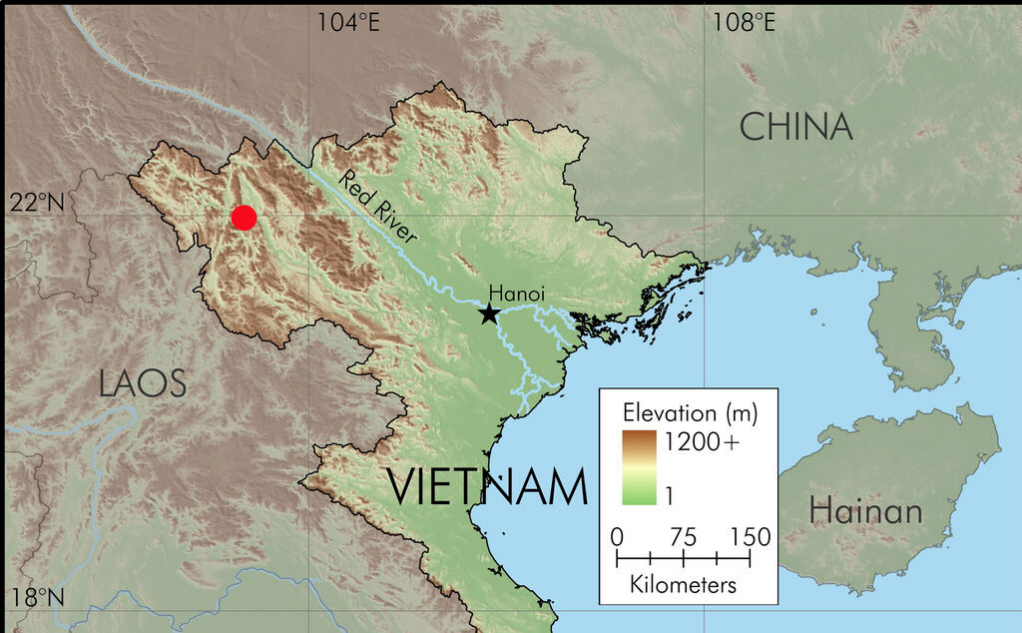
Map showing the type locality (red circle) of *Leptobrachellahuynhi* sp. nov. in Lai Chau Province, northern Vietnam. Black star: Hanoi Capital.

Field surveys were conducted in Sin Ho District, Lai Chau Province, Vietnam (Fig. 1) in July 2016 by T.T. Nguyen, H.T. Ninh, C.V. Hoang and in June 2024 by C.V. Hoang, C.T. Pham, T.Q. Phan, Q.H. Do. Geographic coordinates and elevation were obtained by using a Garmin GPSMAP 78s. After being photographed in life, frogs were anaesthetised and euthanised in a closed vessel with a piece of cotton wool containing ethyl acetate (Simmons 2002), fixed in 80% ethanol for five hours and then later transferred to 70% ethanol for permanent storage. Tissue samples from the thigh muscle were preserved separately in 70% ethanol prior to fixation. Sex was determined by direct observation of calling males in life and by gonadal dissection. Voucher specimens were subsequently deposited in the collection of the Institute of Ecology and Biological Resources (IEBR), Hanoi, Vietnam.

**Molecular phylogenetic analysis**

Tissue samples were extracted using PureLink™ RNA Micro Scale Kit (Thermo Fisher Scientific company), following the manufacturer’s instructions. A fragment of the 16S rRNA mitochondrial gene (~ 560 base pairs) was amplified (Suppl. material 1) that was used recently for *Leptobrachella* (Hoang et al. 2019). Total DNA was amplified using PCR Applied Biosystems, PCR volume consisted of 25 μl, including 12 μl of Mastermix, 6 μl of water, 1 μl of each primer at a concentration of 10 pmol/μl and 5 μl of DNA. Primers used in PCR and sequencing were as follows: LR–N–13398 (5’–CGCCTGTTTACCAAAAACAT –3’; forward) and LR–J 12887 (5’–CCGGTCTGAACTCAGATCACGT –3’; reverse) (Simon et al. 1994). PCR conditions: 94°C for 5 minutes of initial denaturation; with 35 cycles of denaturation at 94°C for 30 s, annealing at 56°C for 30 s and extension at 72°C for 45 s; and the final extension at 72°C for 7 minutes. PCR products were sent to Apical Scientific company for sequencing (https://apicalscientific.com). The obtained sequences were deposited in GenBank under the accession numbers PQ492153–492156. In addition to the four newly-collected samples from Lai Chau Province, we used 107 available sequences of 16S rRNA fragments of 86 morphologically similar species in the genus *Leptobrachella* from GenBank (Hoang et al. 2019, Luong et al. 2023) for phylogenetic analyses. Sequences of Leptobrachiumcf.chapaense and *Megophrystruongsonensis* were included in the analysis as outgroup (Hoang et al. 2019, Luong et al. 2023). Locality information and accession numbers for all sequences included in the analysis can be found in Suppl. material 1.

Chromas Pro software (Technelysium Pty Ltd., Tewantin, Australia) was used to edit the sequences, which were then aligned using the ClustalW (Thompson et al. 1997) option in MEGA11 (Tamura et al. 2021) with default parameters and subsequently optimised manually in BioEdit 7.0.5.2 (Hall 1999). Pairwise comparisons of uncorrected sequence divergences (p-distance) were calculated with MEGA11 (Tamura et al. 2021) where the outgroup was excluded. Variance was estimated using bootstrap method with 1000 replicates using nucleotide substitution, while gaps/missing data were treated via pairwise deletion.

Prior to Bayesian analyses, the optimum substitution models for 16S rRNA partition were selected using Kakusan 4 (Tanabe 2011), based on the Akaike Information Criterion (AIC). We estimated BI and Bayesian posterior probabilities (BPP) in MrBayes v.3.1.2 (Ronquist and Huelsenbeck 2003). The BI summarised two independent runs of four Markov Chains for 10,000,000 generations. A tree was sampled every 100 generations and a consensus topology was calculated for 70,000 trees after discarding the first 30001 trees (burn-in = 3,000,000). We checked parameter estimates and convergence using Tracer version 1.7.1 (Rambaut et al. 2018). Regarding the ML tree inference, IQ-TREE version 1.6.12 (Nguyen et al. 2015) was used with 10,000 ultrafast bootstrap replications (UFB) (Hoang et al. 2018). We considered Bayesian posterior probability (BPP) and ultrafast bootstrap (UFB) support values of greater than or equal to 0.95 to indicate strong support (Felsenstein 1985, Hoang et al. 2018).

**Morphological comparisons**

Measurements were taken from four preserved specimens using a digital calliper to the nearest 0.1 mm (Table 1). The following morphological characteristics were used: Snout-vent length (SVL); head length, from tip of snout to rear of jaws (HL); head width at commissure of jaws (HW); snout length from tip of snout to anterior corner of eye (SNT); diameter of exposed portion of eyeball (ED); interorbital distance (IOD); horizontal diameter of tympanum (TD); distance from anterior edge of tympanum to posterior corner of eye (TED); tibia length with hind-limb flexed (TIB); distance from nostril to anterior edge of eye (EN); distance between nostrils (IN); distance from nostril to tip of snout (NS); manus length from tip of third digit to proximal edge of inner palmar tubercle (ML); pes length from tip of fourth toe to proximal edge of the inner metatarsal tubercle (PL); and length of fingers 1–3 from tip to distal edge of the inner palmar tubercle (F1–3).

## Data resources

All the sequences in this study were retrieved from GenBank and the accession numbers of the newly-determined sequences are shown in Suppl. material 1.

## Taxon treatments

### Leptobrachella huynhi

Hoang, Luong, Nguyen, Nguyen, Ninh, Le, Ziegler & Pham sp. nov.

CF616D91-D262-51FC-939A-5D1320D42374

ED31A8BC-468B-48BF-A2A4-14452CE12467

#### Materials

- **Type status:** Holotype. **Occurrence:** catalogNumber: IEBR A.5213; individualCount: 1; sex: female; lifeStage: adult; occurrenceID: ADA40C4B-F74B-57C6-9C9F-EBEEB882CF34; **Taxon:** scientificNameID: *Leptobrachellahuynhi*; scientificName: *Leptobrachellahuynhi*; class: Amphibia; order: Anura; family: Megophryidae; genus: Leptobrachella; specificEpithet: *huynhi*; **Location:** country: Vietnam; countryCode: VN; stateProvince: Lai Chau; locality: Sin Ho District; verbatimElevation: 1630 m; verbatimLatitude: 22°20'15.4"N; verbatimLongitude: 103°14'32.6"E; verbatimCoordinateSystem: WGS84; **Event:** eventDate: July 13, 2016; eventRemarks: collected by T. T. Nguyen, H. T. Ninh, and C. V. Hoang; **Record Level:** collectionCode: Amphibia; basisOfRecord: PreservedSpecimen
- **Type status:** Paratype. **Occurrence:** catalogNumber: IEBR A.5214; individualCount: 1; sex: female; lifeStage: adult; occurrenceID: 0C7AD5AD-FC31-538D-B0AC-8D2A6E35637D; **Taxon:** scientificNameID: *Leptobrachellahuynhi*; scientificName: *Leptobrachellahuynhi*; class: Amphibia; order: Anura; family: Megophryidae; genus: Leptobrachella; specificEpithet: *huynhi*; **Location:** country: Vietnam; countryCode: VN; stateProvince: Lai Chau; locality: Sin Ho District; verbatimElevation: 1630 m; verbatimLatitude: 22°20'15.4"N; verbatimLongitude: 103°14'32.6"E; verbatimCoordinateSystem: WGS84; **Event:** eventDate: July 13, 2016; eventRemarks: collected by T. T. Nguyen, H. T. Ninh, and C. V. Hoang; **Record Level:** collectionCode: Amphibia; basisOfRecord: PreservedSpecimen
- **Type status:** Paratype. **Occurrence:** catalogNumber: IEBR A.5215; individualCount: 1; sex: female; lifeStage: adult; occurrenceID: CCA446DE-880F-563F-B041-75B83DF3DC56; **Taxon:** scientificNameID: *Leptobrachellahuynhi*; scientificName: *Leptobrachellahuynhi*; class: Amphibia; order: Anura; family: Megophryidae; genus: Leptobrachella; specificEpithet: *huynhi*; **Location:** country: Vietnam; countryCode: VN; stateProvince: Lai Chau; locality: Sin Ho District; verbatimElevation: 1630 m; verbatimLatitude: 22°20'15.4"N; verbatimLongitude: 103°14'32.6"E; verbatimCoordinateSystem: WGS84; **Event:** eventDate: July 13, 2016; eventRemarks: collected by T. T. Nguyen, H. T. Ninh, and C. V. Hoang; **Record Level:** collectionCode: Amphibia; basisOfRecord: PreservedSpecimen
- **Type status:** Paratype. **Occurrence:** catalogNumber: IEBR A.5216; individualCount: 1; sex: female; lifeStage: adult; occurrenceID: E3AA48BF-E1CF-5EA0-853D-8A8090B9706C; **Taxon:** scientificNameID: *Leptobrachellahuynhi*; scientificName: *Leptobrachellahuynhi*; class: Amphibia; order: Anura; family: Megophryidae; genus: Leptobrachella; specificEpithet: *huynhi*; **Location:** country: Vietnam; countryCode: VN; stateProvince: Lai Chau; locality: Sin Ho District; verbatimElevation: 1630 m; verbatimLatitude: 22°20'15.4"N; verbatimLongitude: 103°14'32.6"E; verbatimCoordinateSystem: WGS84; **Event:** eventDate: June 10, 2024; eventRemarks: collected by C. V. Hoang, C. T. Pham, T. Q. Phan, and Q. H. Do; **Record Level:** collectionCode: Amphibia; basisOfRecord: PreservedSpecimen

#### Description

**Figure 2.**
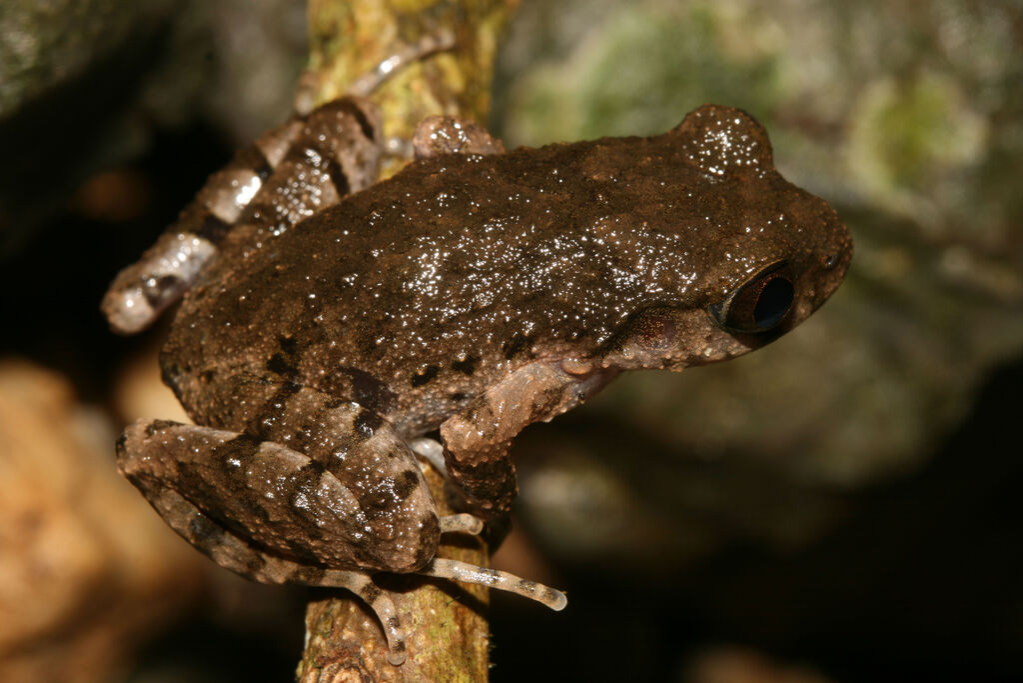
Holotype of *Leptobrachellahuynhi* sp. nov. (IEBR A.5213, female) in life.

**Description of holotype**: Habitus stocky, size medium (SVL 37.8 mm), head longer than wide (HL/HW 1.04); snout slightly projecting beyond margin of lower jaw, obtusely pointed in dorsal view; nostril round, located closer to snout tip than to eye (NS/EN 0.73); canthus rostralis distinct; loreal region sloping; eye diameter shorter than snout length (ED/SNT 0.96); pupil vertical; tympanum distinct, round, tympanum diameter smaller than eye diameter (TD/ED 0.53); slightly concave, tympanic rim not elevated to skin of temporal region; pineal ocellus absent; vomerine teeth absent; tongue large, broad, slightly concave at tip; supratympanic ridge slightly rough with few small nodules; supratympanic fold forming a distinct ridge, running from posterior corner of eye towards supra-axillary gland (Fig. 2).

**Figure 3.**
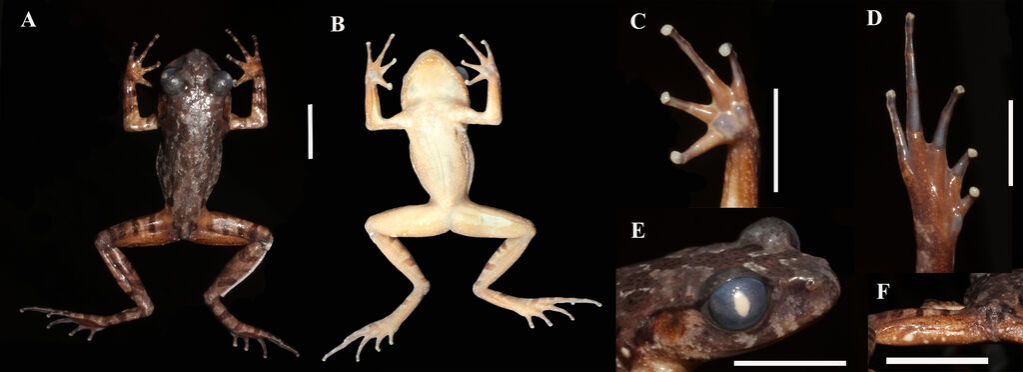
Holotype of *Leptobrachellahuynhi* sp. nov. (IEBR A.5213), **A** dorsal view; **B** ventral view; **C** left hand; **D** right foot; **E** right side of head; **F** cloacal region in preservative.

Fore-limbs slender; finger tips round, slightly broader than phalange width; finger webbing absent, lateral fringes narrow; relative finger lengths: II < I < IV < III; nuptial pad absent; subarticular tubercles absent, replaced by distinct dermal ridges; a large, round inner palmar tubercle, distinctly separated from small, laterally compressed outer palmar tubercle (Fig. 3).

Hind-limbs slender, tibia length approximately half of snout-vent length (TIB/SVL 0.49). Tips of toes round, broader than phalange width; relative toe lengths: I <II < V < III < IV; interdigital toe webbing absent; toes with narrow lateral fringes; subarticular tubercles absent, replaced by distinct dermal ridges; inner metatarsal tubercle small, oval, pronounced, outer metatarsal tubercle absent (Fig. 3).

Skin texture in life. Skin on entire dorsum shagreened with low, round tubercles of irregular sizes, alternately arranged and scattered, tubercles becoming smaller towards venter; ventral skin smooth; pectoral gland oval, 1.6 mm in diameter; supra-axillary gland raised, oval, 0.6 mm in diameter; femoral glands round, smaller than pectoral gland (~ 0.8 mm in diameter), located on posteroventral surfaces of thighs, closer to knee than to vent; ventrolateral glands present, dorsolaterally compressed, forming an incomplete line (Fig. 2).

Colour in life. Dorsum grey-brown with indistinct dark brown markings, flank and heel light-brown with some dark flecks; inter-orbital region with a stacking double Y-shaped marking, anterior part stretched to two upper lips, posterior part stretched towards the area between axillae; marbling between axillae and inguinal region; tympanum with brown colour that blends well with the surrounding region, a dark brown stripe below supratympanic ridge, running from posterior corner of eye towards supra-axillary gland; supra-axillary region light-brown; dorsal surface of limbs, fingers and toes with diffuse, transverse dark brown bars, interwoven by brown bars; centre of belly creamy-white, outer edges of belly brown with small whitish spots; ventral surface of the chin, thighs, arms and tibiotarsus brown with small whitish spots; femoral, pectoral and dorsolateral glands creamy-white; iris copper (Fig. 2).

Colour in preservative. Dorsal surface grey; markings dark-grey, edged in white-grey; dorsal surface of limbs, fingers and toes with diffuse, transverse dark-grey bars, interwoven by beige bars; centre of belly, throat and chest cream; chin, thighs, arms, tibiotarsus and outer edges of belly, throat and chest beige with small cream spots; cream pectoral glands became indistinct in preservative (Fig. 3).

#### Diagnosis

*Leptobrachellahuynhi* sp. nov. is distinguished from its congeners by a combination of the following morphological characteristics: Size medium (SVL 37.8–40.2 mm, n = 4 adult females); head longer than wide; tympanum distinct; skin on entire dorsum shagreened; toes without interdigital webbing and with narrow lateral fringes; supratympanic ridge slightly rough with few nodules; dorsum grey-brown with indistinct dark brown markings; interorbital region with a stacking double Y-shaped marking; centre of belly creamy-white, outer edges of belly brown with small whitish spots; iris copper. In addition, the new species is genetically distinct from other species in the genus with uncorrected genetic distances ≥ 3.62% (mitochondrial gene 16S rRNA).

#### Etymology

The new species is named after Prof. Dr. Huynh Huy Dang, Chairman of the Zoological Society of Vietnam, to honour his great contributions to the vertebrate fauna of Vietnam. We recommend “Huynh’s Leaf-litter Frog” as the common English name and “Cóc mày huỳnh” as the Vietnamese name.

#### Distribution

*Leptobrachellahuynhi* sp. nov. is currently known from Sin Ho District, Lai Chau Province, Vietnam (Fig. 1).

#### Ecology

**Figure 4.**
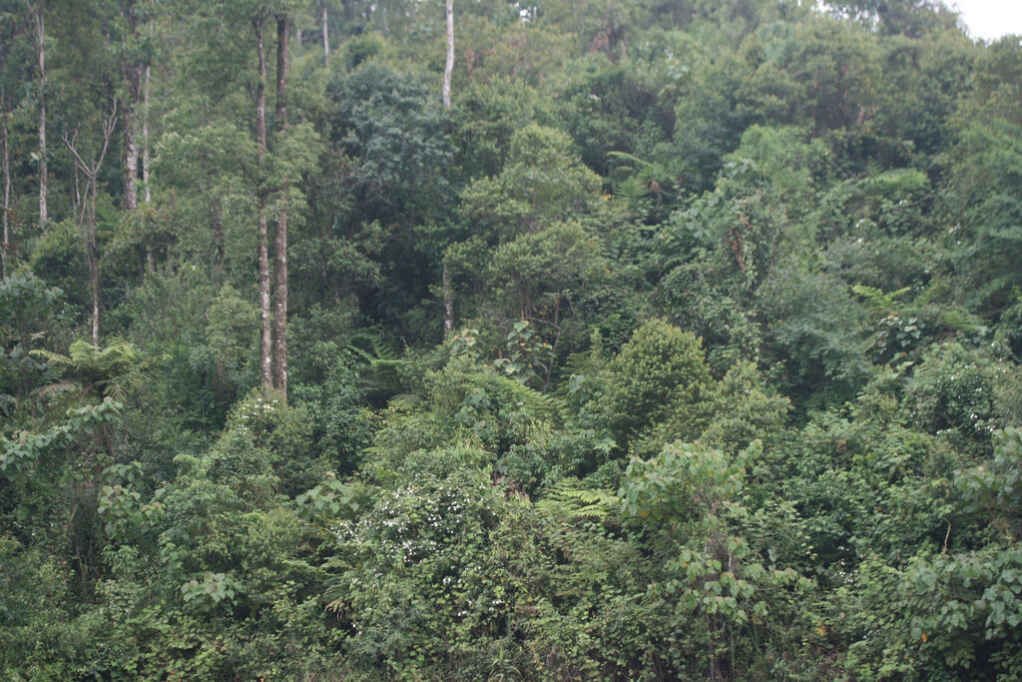
Habitat of *Leptobrachellahuynhi* sp. nov. in Sin Ho District, Lai Chau Province, Vietnam.

Specimens of the new species were found in small streams at elevations ~ 1630 m a.s.l. in evergreen forest nearby Sin Ho Town and intercity road DT128 (Fig. 4). *Leptobrachellahuynhi* sp. nov. occurs sympatrically with *L.ventripunctata*.

#### Notes

##### Variation

Type specimens vary in body size and colour pattern in life (Table 1, Figs 2, 3). Glands around cloacal opening vary in size and number. In preservative, dorsal skin texture varies from finely tuberculate to almost smooth.

#### Comparisons

Comparative morphological data of *Leptobrachellahuynhi* sp. nov. and 79 recognised *Leptobrachella* species occurring north of the Isthmus of Kra are listed in Suppl. material 2.

**Figure 5.**
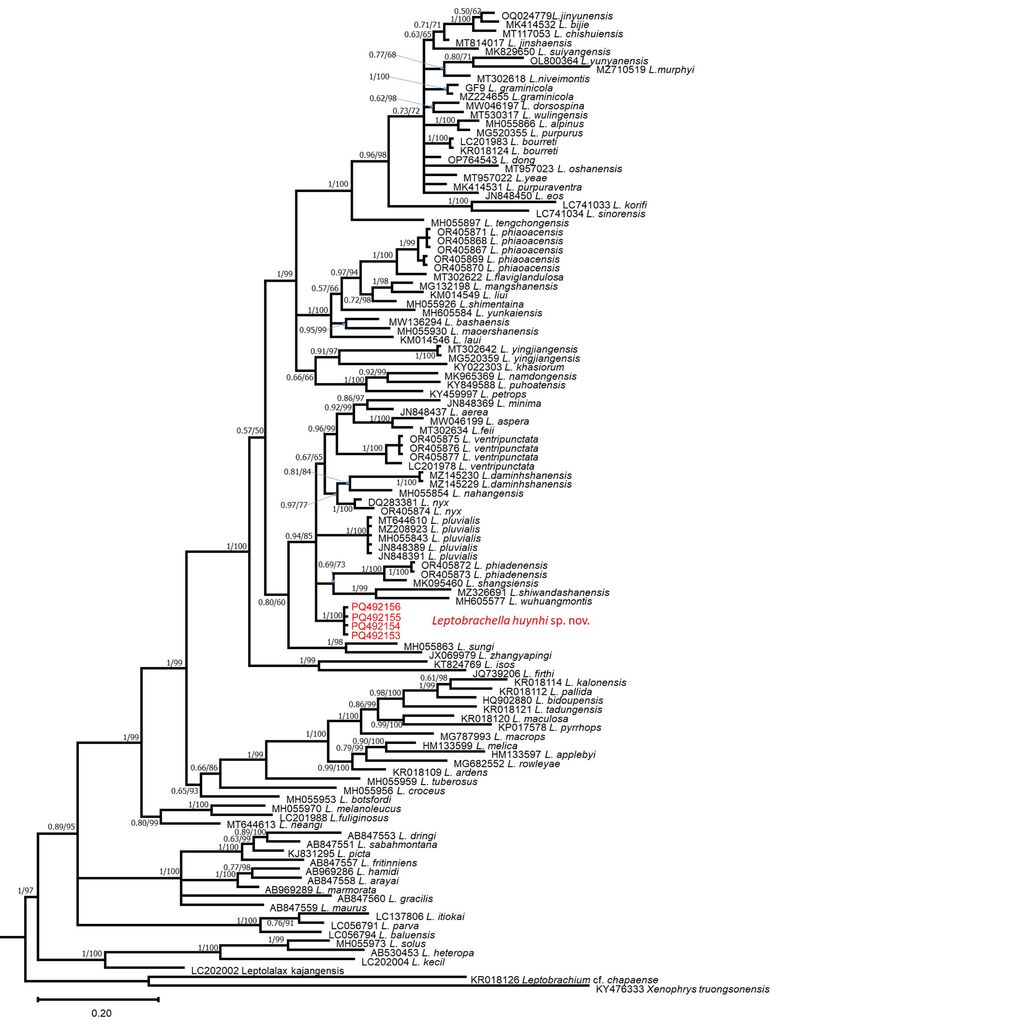
The Bayesian Inference (BI) tree, based on the partial 16S rRNA mitochondrial gene. Values at nodes correspond to BI/ML support values, respectively. Leptobrachiumcf.chapaense and *Megophrystruongsonensis* are used in the outgroup.

In the phylogenetic tree (Fig. 5), *Leptobrachellahuynhi* sp. nov. is a sister taxon to *L.shiwandashanensis*, *L.wuhuangmontis*, *L.shangsiensis*, *L.pluvialis*, *L.minima*, *L.ventripunctata*, *L.aerea*, *L.feii*, *L.aspera*, *L.damingshanensis*, *L.nahangensis*, *L.nyx* and *L.phiadenensis* with a high support value (0.94 in BI, 85 in ML) and the new species can be distinguished from them by genetic divergences of at least 3.62% (Suppl. material 3).

Morphologically, the new species differs from *L.shiwandashanensis* by having a larger body size in females (SVL 37.8–40.2 mm vs. 32.3–35.9 mm in *L.shiwandashanensis*), toes with narrow lateral fringes (vs. absent in *L.shiwandashanensis*), head longer than wide (HL/HW 1.05 vs. 0.95 in *L.shiwandashanensis*), a greater ratio of HL/SVL (0.39 vs. 0.32 in *L.shiwandashanensis*), a greater ratio of TIB/SVL (0.49 vs. 0.43 in *L.shiwandashanensis*), tympanum diameter larger than half of eye diameter (TD/ED 0.61 vs. 0.49 in *L.shiwandashanensis*) and iris copper (vs. iris bicoloured: upper half brownish-red and silver in the lower half in *L.shiwandashanensis*) (Chen et al. 2021, Lo et al. 2022); from *L.wuhuangmontis* by having a larger body size in females (SVL 37.8–40.2 mm vs. 33.0–36.0 mm in *L.wuhuangmontis*), dorsal skin shagreened with low, round tubercles (vs. rough, scattered with dense conical tubercles in *L.wuhuangmontis*), centre of belly creamy-white, outer edges of belly brown with small whitish spots (vs. belly greyish-white mixed by tiny white and black dots in *L.wuhuangmontis*), a greater ratio of HL/SVL (0.39 vs. 0.36 in *L.wuhuangmontis*), a greater ratio of TIB/SVL (0.49 vs. 0.45 in *L.wuhuangmontis*) and iris copper (vs. iris bicoloured: coppery yellow on upper half and silver on lower half in *L.wuhuangmontis*) (Wang et al. 2018); from *L.shangsiensis* by having a larger body size in females (SVL 37.8–40.2 mm vs. 30.8–35.9 mm in *L.shangsiensis*), dorsal skin with low, round tubercles (vs. mostly smooth with numerous tiny tubercles in *L.shangsiensis*), centre of belly creamy-white, outer edges of belly brown with small whitish spots (vs. yellowish-creamy-white in *L.shangsiensis*), head longer than wide (HL/HW 1.05 vs. 0.92 in *L.shangsiensis*), a greater ratio of HL/SVL (0.39 vs. 0.31 in *L.shangsiensis*) , a greater ratio of TIB/SVL (0.49 vs. 0.47 in *L.shangsiensis*), tympanum diameter larger than half of eye diameter (TD/ED 0.61 vs. 0.50 in *L.shangsiensis*) and iris copper (vs. iris copper in the upper and silver in the lower fifth in *L.shangsiensis*) (Chen et al. 2019); from *L.pluvialis* by having a larger body size in females (SVL 37.8–40.2 mm vs. 25.5–33.5 mm in *L.pluvialis*), dorsal skin shagreened with low, round tubercles (vs. smooth, flattened dorsal tubercles on dorsal in *L.pluvialis*), toes with narrow lateral fringes (vs. without lateral fringes in *L.pluvialis*), centre of belly creamy-white, outer edges of belly brown with small whitish spots (vs. dirty white with dark brown marbling in *L.pluvialis*), a greater ratio of NS/EN (0.81 vs. 0.78 in *L.pluvialis*) and iris copper (vs. dark golden in *L.pluvialis*) (Ohler et al. 2000, Nguyen et al. 2021); from *L.minima* by having a larger body size in females (SVL 37.8–40.2 mm vs. 31.6–37.3 mm in *L.minima*), skin on entire dorsum shagreened with low, round tubercles (vs. smooth in *L.minima*), toes with narrow lateral fringes (vs. without lateral fringes in *L.minima*), centre of belly creamy-white, outer edges of belly brown with small whitish spots (vs. creamy-white in *L.minima*), toe webbing absent (vs. toe webbing rudimentary in *L. L.minima*) and iris copper (vs. iris dark gold above and grey below in *L.minima*) (Taylor 1962, Ohler et al. 2011); from *L.ventripunctata* by having a larger body size in females (SVL 37.8–40.2 mm vs. 31.5–35.0 mm *L.ventripunctata*), skin on entire dorsum shagreened with low, round tubercles (vs. longitudinal skin ridges in *L.ventripunctata*), toes with narrow lateral fringes (vs. without lateral fringes in *L.ventripunctata*), toes webbing absent (vs. rudimentary toes webbing in *L.ventripunctata*), centre of belly creamy-white, outer edges of belly brown with small whitish spots (vs. chest and belly with dark brown spots in *L.ventripunctata*) and iris copper (vs. bicoloured iris: copper above and grey-brown below in *L.ventripunctata*) (Fei et al. 1991, Fei et al. 2009, Fei et al. 2012); from *L.aerea* by having toes with narrow lateral fringes (vs. well developed in *L.aerea*), variable dorsolateral markings (vs. indistinct dorsolateral markings in *L.aerea*), toes with narrow lateral fringes (vs. well developed in *L.aerea*), a greater ratio of HL/SVL (0.39 vs. 0.37 in *L.aerea*) and a smaller ratio of ED/SNT (0.88 vs. 0.91 in *L.aerea*) (Rowley et al. 2010); from *L.feii* by having a larger body size in females (SVL 37.8–40.2 mm vs. 25.7 mm in *L.feii*), centre of belly creamy-white, outer edges of belly brown with small whitish spots (vs. creamy-white with black blotches in *L.feii*), ventrolateral glands forming a discontinuous line (vs. ventrolateral glands forming a continuous line in *L.feii*), a smaller ratio of ED/SNT (0.88 vs. 0.92 in *L.feii*), a greater ratio of TIB/SVL (0.49 vs. 0.46 in *L.feii*) and iris copper (vs. iris bicoloured: golden orange in upper half and silver white in lower half in *L.feii*) (Chen et al. 2020); from *L.aspera* by having a larger body size in females (SVL 37.8–40.2 mm vs. 25.0–26.4 mm in *L.aspera*), skin on entire dorsum shagreened with low, round tubercles (vs. rough with dense conical granules, tubercles and dermal ridges in *L.aspera*), centre of belly creamy-white, outer edges of belly brown with small whitish spots (vs. creamy-white with distinct dark spots and distinct regular dark patches on skin of chest and abdomen in *L.aspera*), a greater ratio of HL/SVL (0.39 vs. 0.33–0.35 in *L.aspera*), relative finger lengths II <I < IV < III (vs. I < IV < II < III in *L.aspera*) and iris copper (vs. iris bicoloured: amber on upper half and silver on lower half in *L.aspera*) (Wang et al. 2020); from *L.damingshanensis* by having skin on entire dorsum shagreened with low, round tubercles (vs. rough with small, raised tubercles and ridges in *L.damingshanensis*), variable dorsolateral markings (vs. indistinct dorsolateral markings in *L.damingshanensis*), a greater ratio of HL/HW (1.05 vs. 0.89 in *L.damingshanensis*), a greater ratio of HL/SVL (0.39 vs. 0.31 in *L.damingshanensis*), a smaller ratio of ED/SNT (0.88 vs. 0.92 in *L.damingshanensis*) and iris copper (vs. iris bicoloured upper half copper, fading to silver in lower half in *L.damingshanensis*) (Chen et al. 2021b); from *L.nahangensis* by having skin on entire dorsum shagreened with low, round tubercles (vs. smooth in *L.nahangensis*), centre of belly creamy-white, outer edges of belly brown with small whitish spots (vs. creamy-white with light speckling on throat and chest in *L.nahangensis*) and iris copper (vs. iris gold, uniformly distributed with minute black reticulations in *L.nahangensis*) (Lathrop et al. 1998); from *L.nyx* by having ventrolateral glands forming a discontinuous line (vs. lateroventral glandular ridge poorly distinct in *L.nyx*), toes with narrow lateral fringes (vs. absent lateral fringes in *L.nyx*), a greater ratio of HL/SVL (0.39 vs. 0.38 in *L.nyx*), a greater ratio of TIB/SVL (0.49 vs. 0.48 in *L.nyx*) and skin on entire dorsum shagreened with low, round tubercles of irregular sizes, alternately arranged and scattered, tubercles becoming smaller towards ventral (vs. dorsal and lateral parts of head and body: snout and side of head smooth; region between eyes, back and flanks with flat glandular warts, quite indistinct on dorsal part in *L.nyx*) (Ohler et al. 2011); and from *L.phiadenensis* by having a larger body size in females (SVL 37.8–40.2 mm vs. 27.6–28.6 mm in *L.phiadenensis*), a greater ratio of (HL/HW 1.05 vs. 0.94–0.97 in *L.phiadenensis*), a greater ratio of HL/SVL (0.39 vs. 0.35 in *L.phiadenensis*) and iris copper (vs. iris bicoloured: copper in upper half, fading to silvery grey in its lower half in *L.phiadenensis*) (Luong et al. 2023).

## Analysis

**Phylogenetic analysis**

This analysis involved 109 sequences on the 16S rRNA dataset. Codon positions included were 1st+2nd+3rd+Noncoding. There was a total of 581 positions in the final dataset, 268 sites were conserved and 300 sites were variable, of which 238 were found to be potentially parsimony-informative. Evolutionary analyses were conducted in MEGA11 (Tamura et al. 2021). The overall Transition/Transversion bias was R = 1.85. Substitution patterns and rates were estimated under the Tamura (1992) model. The nucleotide frequencies were A = 30.52%, T/U = 24.94%, C = 24.04% and G = 20.49%. The ML and Bayesian analyses produced topologies with –ln *L* = 10054.295 and 10118.6703, respectively. Phylogenetic analyses employing ML and BI methods were nearly identical, with most well-supported nodes on the ML tree also well supported on the Bayesian tree and only the BI tree is presented (Fig. 5). In both analyses, four newly-collected samples are genetically sister to the closest clade with strong nodal support from both analyses (0.94/85) (Fig. 5), including 13 species *L.shiwandashanensis* Chen, Peng, Pan, Liao, Liu & Huang; *L.wuhuangmontis* Wang, Yang & Wang; *L.shangsiensis* Chen, Liao, Zhou & Mo; *L.pluvialis* Ohler, Marquis, Swan & Grosjean; *L.minima* Taylor; *L.ventripunctata* Fei, Ye & Li; *L.aerea* Rowley, Stuart, Richards, Phimmachak & Sivongxay; *L.feii* Chen, Yuan & Che; *L.aspera* Wang, Lyu, Qi & Wang; *L.damingshanensis* Chen, Yu, Cheng, Meng, Wei, Zhou & Lu; *L.nahangensis* Lathrop, Murphy, Orlov & Ho; *L.nyx* Ohler, Wollenberg, Grosjean, Hendrix, Vences, Ziegler & Dubois; and *L.phiadenensis* Luong, Hoang, Pham, Ziegler & Nguyen.

Interspecific uncorrected p-distance of the analysed taxa ranged from 1.65% (between *Leptobrachellajinyunensis* Shi, Shen, Wang, Jiang & Wang and *L.bijie* Wang, Li, Li, Chen & Wang) to 26.25% (between *L.laui* Sung, Yang & Wang and *L.sola* Matsui). However, uncorrected p-distance of *L.ventripunctata* between two populations from Phongsaly Province (Laos) and Cao Bang Province (Vietnam) reached up to 1.68% (Suppl. material 3). Moreover, the genetic divergence of four newly-collected samples and their congeners ranged from 3.62% (*L.nyx*) to 18.51% (*L.gracilis*), indicating that the differentiations between four newly-collected samples and their congeners had reached specific level.

Based on the distinct molecular divergence in concert with diagnostic morphological differences compared to congeners, a *Leptobrachella* population from Lai Chau Province of Vietnam is described as a new species.

## Discussion

Our phylogenetic analyses of the *Leptobrachella* species correspond well with previous studies, for example Rowley et al. (2017), Chen et al. (2018), Hoang et al. (2019) and Luong et al. (2023). In terms of genetical divergence, *Leptobrachellahuynhi* sp. nov. is separated from the sympatric species, *L.ventripunctata*, by p-distances of 6.01–6.22% and *L.nyx*, by p-distances of 3.62% (16S gene). In the *L.ventripunctata* group, both BI and ML trees showed a clear separation of two populations from Phongsaly Province (Laos) and Cao Bang Province (Vietnam) with a high support value (1 in BI, 100 in ML). Moreover, uncorrected p-distance of *L.ventripunctata* between two populations was up to 1.68%. This shows that the *L.ventripunctata* group is a complex species group that needs to be studied more closely in the future. In terms of morphology, *Leptobrachellahuynhi* sp. nov. is separated from the sympatric species (*L.ventripunctata* and *L.nyx*) by having a larger body size in females (SVL 37.8–40.2 mm), skin on entire dorsum shagreened with low, round tubercles, toes with narrow lateral fringes, toes webbing absent, centre of belly creamy-white, outer edges of belly brown with small whitish spots, iris copper, ventrolateral glands forming a discontinuous line, toes with narrow lateral fringes, a greater ratio of HL/SVL and a greater ratio of TIB/SVL

In terms of conservation concern, the new species was found in evergreen forest near Sin Ho Town and the area is not located in any protected areas. The habitat of *Leptobrachellahuynhi* sp. nov. is under the risk of degradation by the extension of agricultural land and infrastructure development of Sin Ho Town as well as by increasing human travel on intercity road DT128. The discovery of *Leptobrachellahuynhi* sp. nov. brings the total number of known species in the genus to 103 and the species number known from Vietnam to 35 (Luong et al. 2023). Further fieldwork likely will help to uncover additional new taxa of the genus in Vietnam, particularly in remote montane forests.

## Supplementary Material

XML Treatment for Leptobrachella huynhi

BDF9970B-B7DC-52C9-8A8B-EF6ACDFE6618 10.3897/BDJ.12.e136491.suppl1 Supplementary material 1 GenBank accession numbers and associated samples Data type GenBank numbers and associated samples Brief description GenBank accession numbers and associated samples used in this study. File: oo_1121564.docx https://binary.pensoft.net/file/1121564 Chung Van Hoang, Anh Mai Luong, Truong Quang Nguyen, Tao Thien Nguyen, Hoa Thi Ninh, Linh Hoang Tu Le, Thomas Ziegler, Cuong The Pham

8B33542F-263D-56BA-9A7F-D6FB172D884C 10.3897/BDJ.12.e136491.suppl2 Supplementary material 2 Diagnostic characters on morphology Data type morphological Brief description Selected diagnostic characters for the species in the genus *Leptobrachella* occurring north of the Isthmus of Kra (modified from Rowley et al. (2017); Yuan et al. (2017); Nguyen et al. (2018); Wang et al. (2018); Liu et al. (2023); Luong et al. (2023). NA: Not available. File: oo_1121561.docx https://binary.pensoft.net/file/1121561 Chung Van Hoang, Anh Mai Luong, Truong Quang Nguyen, Tao Thien Nguyen, Hoa Thi Ninh, Linh Hoang Tu Le, Thomas Ziegler, Cuong The Pham

5A5C1276-3BD8-5D67-8F81-E33A9B7D4A87 10.3897/BDJ.12.e136491.suppl3 Supplementary material 3 Uncorrected (“p”) distance matrix Data type phylogenetic Brief description Uncorrected (“p”) distance matrix showing percentage pair-wise genetic divergence (16S gene) between analysed members of the *Leptobrachella* species. File: oo_1121562.docx https://binary.pensoft.net/file/1121562 Chung Van Hoang, Anh Mai Luong, Truong Quang Nguyen, Tao Thien Nguyen, Hoa Thi Ninh, Linh Hoang Tu Le, Thomas Ziegler, Cuong The Pham

## Figures and Tables

**Table 1.** Measurements (in mm) and proportions of the type series of *Leptobrachellahuynhi* sp. nov. (H = Holotype, P = Paratype, Min = minimum, Max = maximum, SD = standard deviation for other abbreviations see Material and Methods).

	*Leptobrachellahuynhi* sp. nov.				Min–Max	TB±SD
Sex	Female					
	**IEBR A.5213**	**IEBR A.5214**	**IEBR A.5215**	**IEBR A.5216**		
Type	H	P	P	P		
SVL	37.8	39.9	39.9	40.2	37.8–40.2	39.5 ± 1.1
HL	14.7	16.0	15.5	15.8	14.7–16	15.5 ± 0.5
HW	14.2	15.5	15.0	14.5	14.2–15.5	14.8 ± 0.6
SNT	5.9	6.4	6.3	5.7	5.4–6.4	5.9 ± 0.5
ED	5.7	5.3	4.7	5.0	4.7–5.7	5.2 ± 0.4
IOD	4.7	5.0	5.0	4.4	4.4–5	4.8 ± 0.3
TD	3.0	3.3	3.0	3.3	3–3.3	3.1 ± 0.1
TED	2.1	2.1	2.3	2.3	2.1–2.3	2.2 ± 0.1
TIB	18.6	19.4	19.8	18.9	18.6–19.8	19.2 ± 0.5
EN	3.4	3.9	3.6	3.4	3.4–3.9	3.6 ± 0.2
IN	3.7	4.0	3.9	3.5	3.5–4	3.8 ± 0.2
NS	2.5	2.8	3.6	2.6	2.5–3.6	2.9 ± 0.5
ML	11.1	11.2	11.8	11.3	11.1–11.8	11.3 ± 0.3
PL	18.2	19.8	19.2	18.2	18.2–19.8	18.9 ± 0.8
F1	5.4	5.0	5.7	5.4	5–5.7	5.4 ± 0.3
F2	5.1	4.9	5.2	4.2	4.2–5.2	4.8 ± 0.5
F3	7.9	8.5	7.7	8.1	7.7–8.5	8 ± 0.3
HL/HW	1.04	1.03	1.03	1.08	1.03–1.08	1.05 ± 0.03
HL/SVL	0.39	0.40	0.39	0.39	0.39–0.4	0.39 ± 0.01
TIB/SVL	0.49	0.49	0.50	0.47	0.47–0.5	0.49 ± 0.01
TD/ED	0.53	0.61	0.64	0.65	0.53–0.65	0.61 ± 0.06
ED/SNT	0.96	0.84	0.75	0.88	0.75–1.05	0.88± 0.13
NS/EN	0.73	0.72	1.02	0.76	0.72–1.02	0.81 ± 0.14
