# Supplementary material for: A new species of Leptobrachella Smith 1925 (Anura, Megophryidae) from Lai Chau Province, Vietnam
Source: Biodivers Data J. 2024 Nov 4;12:e136491. doi: 10.3897/BDJ.12.e136491 (PMC11555430; doi:10.3897/BDJ.12.e136491)
Supplement: Supplementary material 1 — GenBank accession numbers and associated samples [file bdj-12-e136491-s001.docx]

**Suppl. material 1.** GenBank accession numbers and associated samples were used in this study.

**Author**: Chung Van Hoang, Anh Mai Luong, Truong Quang Nguyen, Tao Thien Nguyen, Hoa Thi Ninh, Linh Hoang Tu Le, Thomas Ziegler, Cuong The Pham

**Data type**: GenBank numbers and associated samples

**Brief description:** GenBank accession numbers and associated samples were used in this study.

|  | **Species** | **Voucher no** | **GenBank no.** | **Locality** | **References** |
| --- | --- | --- | --- | --- | --- |
| 1 | *Leptobrachella huynhi* sp. nov. | IEBR A.5830 | CB15 | Vietnam, Lai Chau Provice, Sin Ho District | This study |
| 2 | *Leptobrachella huynhi* sp. nov. | IEBR A.5827 | CB16 | Vietnam, Lai Chau Provice, Sin Ho District | This study |
| 3 | *Leptobrachella huynhi* sp. nov. | IEBR A.5825 | CB19 | Vietnam, Lai Chau Provice, Sin Ho District | This study |
| 4 | *Leptobrachella huynhi* sp. nov. | IEBR A.5971 | CB21 | Vietnam, Lai Chau Provice, Sin Ho District | This study |
| 5 | *L. aerea* | RH60165 | JN848437 | Vietnam, Quang Binh Province | Ohler et al. 2011 |
| 6 | *L. alpina* | KIZ046816 | MH055866 | China, Yunnan Province | Chen et al. 2018 |
| 7 | *L. applebyi* | AMS R171703 | HM133597 | Vietnam, Quang Binh Province | Rowley et al. 2012 |
| 8 | *L. arayai* | BORNEENSIS 22931 | AB847558 | Malaysia, Borneo | Matsui et al. 2014 |
| 9 | *L. ardens* | VNMN 04707 | KR018109 | Vietnam, Gia Lai Province | Rowley et al. 2015 |
| 10 | *L. aspera* | SYS a007743 | MW046199 | China, Yunnan Province | Wang et al. 2020 |
| 11 | *L. baluensis* | BORN:8595 | LC056794 | Malaysia, Borneo | Eto et al. 2015 |
| 12 | *L. bashaensis* | GIB13196403 | MW136294 | China, Guizhou Province | Lou 2020 |
| 13 | *L. bidoupensis* | AMS R173134 | HQ902880 | Vietnam, Lam Dong Province | Rowley et al. 2011 |
| 14 | *L. bijie* | SYS a007313/CIB110002 | MK414532 | China, Guizhou Province | Wang et al. 2019 |
| 15 | *L. botsfordi* | AMSR176540 | MH055952 | Vietnam, Lai Chau Province | Chen et al. 2018 |
| 16 | *L. bourreti* | AMS R177673 | KR018124 | Vietnam, Lao Cai Province | Rowley et al. 2015 |
| 17 | *L. bourreti* | MNHNP:5659 | LC201983 | Vietnam, Lao Cai Province | Matsui et al. 2017 |
| 18 | *L. chishuiensis* | CIBCS20190518047 | MT117053 | China, Guizhou Province | Li et al. 2020 |
| 19 | *L. crocea* | SYS:a007313 | MH055956 | Vietnam, Quang Nam Province | Chen et al. 2018 |
| 20 | *L. daminhshanensis* | NNU202103281 | MZ145229 | China, Guangxi Province | Chen et al. 2021c |
| 21 | *L. daminhshanensis* | NNU202103282 | MZ145230 | China, Guangxi Province | Chen et al. 2021c |
| 22 | *L. dong* | CIB ZNY2022013 | OP764543 | China, Hunan Province | Liu et al. 2023 |
| 23 | *L. dorsospina* | SYS a004974 | MW046197 | China, Guizhou Province | Wang et al. 2020 |
| 24 | *L. dringi* | KUHE:55610 | AB847553 | Malaysia, Borneo | Matsui et al. 2014 |
| 25 | *L. eos* | MNHN:2004.0278 | JN848450 | Laos, Phongsaly Province | Ohler et al. 2011 |
| 26 | *L. feii* | KIZ048894 | MT302634 | China, Yunnan Province | Chen et al. 2020 |
| 27 | *L. firthi* | AMS R176524 | JQ739206 | Vietnam, Kon Tum Province | Rowley et al. 2012 |
| 28 | *L. flaviglandulosa* | KIZ032627 | MT302622 | China, Yunnan Province | Chen et al. 2020 |
| 29 | *L. fritinniens* | KUHE 55371 | AB847557 | Malaysia, Borneo | Matsui et al. 2014 |
| 30 | *L. fuliginosa* | KUHE:20197 | LC201988 | Thailand, Phetchaburi Province | Matsui et al. 2017 |
| 31 | *L. gracilis* | KUHE 55624 | AB847560 | Malaysia, Borneo | Matsui et al. 2014 |
| 32 | *L. graminicola* | IEBR A.5219 | GF9 | Vietnam, Lao Cai Province | This study |
| 33 | *L. graminicola* | VNMN 010910 | MZ224655 | Vietnam, Lao Cai Province | Nguyen et al. 2021 |
| 34 | *L. hamidi* | KUHE 17545 | AB969286 | Malaysia, Borne Province | Matsui et al. 2014 |
| 35 | *L. heteropus* | KUHE 15487 | AB530453 | Malaysia, Peninsula Province | Matsui et al. 2010 |
| 36 | *L. isos* | VNMN A 2015.4 | KT824769 | Vietnam, Gia Lai Province | Rowley et al. 2015 |
| 37 | *L. itiokai* | KUHE:55898 | LC137806 | Malaysia, Sarawak Province | Eto et al. 2016 |
| 38 | *L. jinshaensis* | CIBJS20200516004 | MT814017 | China, Guizhou Province | Cheng et al. 2021 |
| 39 | *L. jinyunensis* | CIB 119040 | OQ024779 | China, Chongqing City | Shi et al. 2023 |
| 40 | *L. kajangensis* | LSUHC:4439 | LC202002 | Malaysia | Matsui et al. 2017 |
| 41 | *L. kalonensis* | IEBR A.2014.15 | KR018114 | Vietnam, Binh Thuan Province | Rowey et al. 2015 |
| 42 | *L. kecil* | KUHE:52440 | LC202004 | Malaysia | Matsui et al. 2017 |
| 43 | *L. khasiorum* | SDBDU 2009.329 | KY022303 | India, Meghalaya Province | Mahony et al. 2017 |
| 44 | *L. korifi* | KUHE:19134 | LC741033 | Thailand, Doi Inthanon | Matsui et al. 2023 |
| 45 | *L. laui* | SYS A002057 | KM014546 | China, Shenzhen Province | Sung et al. 2014 |
| 46 | *L. liui* | SYS A001620 | KM014549 | China, Jiangxi Province | Sung et al. 2014 |
| 47 | *L. macrops* | ZMMU-A5823 | MG787993 | Vietnam, Phu Yen Province | Duong et al. 2008 |
| 48 | *L. maculosa* | ZFMK 96600 | KR018120 | Vietnam, Ninh Thuan Province | Rowley et al. 2015 |
| 49 | *L. mangshanensis* | MSZTC201703 | MG132198 | China, Hunan Province | Hou et al. 2018 |
| 50 | *L. maoershanensis* | SYSa002898 | MH055930 | China, Guangxi Province | Chen et al. 2018 |
| 51 | *L. marmorata* | ZFMK 96600 | AB969289 | Vietnam, Ninh Thuan Province | Matsui et al. 2014 |
| 52 | *L. maura* | SP 21450 | AB847559 | Malaysia, Borneo | Matsui et al. 2014 |
| 53 | *L. melanoleuca* | KIZ024598 | MH055970 | Thailand | Chen et al. 2018 |
| 54 | *L. melica* | MVZ 258198 | HM133599 | Cambodia, Ratanakiri Province | Rowley et al. 2010b |
| 55 | *L. minima* | K3124 | JN848369 | Thailand, Chiangmai Province | Ohler et al. 2011 |
| 56 | *L. murphyi* | KIZ034039 | MZ710519 | Thailand, Chiangmai Province | Chen et al. 2021a |
| 57 | *L. nahangensis* | ZMMU-NAP-02259 | MH055854 | Vietnam, Tuyen Quang Province | Chen et al. 2018 |
| 58 | *L. namdongensis* | VNUF A.2017.37 | MK965389 | Vietnam, Thanh Hoa Province | Hoang et al. 2019 |
| 59 | *L. neangi* | CBC 1624 | MT644613 | Cambodia, Koh Kong Province | Stuart et al. 2020 |
| 60 | *L. niveimontis* | KIZ015734 | MT302618 | China, Yunnan Province | Chen et al. 2021c |
| 61 | *L. nyx* | AMNH A163810 | DQ283381 | Vietnam, Ha Giang Province | Frost et al. 2006 |
| 62 | *L. nyx* | IEBR A.5222 | OR405874 | Vietnam, Cao Bang Province | Luong et al. 2023 |
| 63 | *L. oshanensis* | SYS A001830 | KM014810 | China, Sichuan Province | Sung et al. 2016 |
| 64 | *L. pallida* | UNS00510 | KR018112 | Vietnam, Lam Dong Province | Rowley et al. 2015 |
| 65 | *L. parva* | KUHE:55308 | LC056791 | Malaysia, Borneo | Eto et al. 2015 |
| 66 | *L. petrops* | VNMN 2016 A.06 | KY459999 | Vietnam, Tuyen Quang Province | Rowley et al. 2017 |
| 67 | *L. picta* | UNIMAS 8705 | KJ831295 | Malaysia, Borneo | Oberhummer et al. 2017 |
| 68 | *L. phiadenensis* | IEBR A.5205 | OR405872 | Vietnam, Cao Bang Province | Luong et al. 2023 |
| 69 | *L. phiadenensis* | IEBR A.5206 | OR405873 | Vietnam, Cao Bang Province | Luong et al. 2023 |
| 70 | *L. phiaoacensis* | IEBR A.5201 | OR405867 | Vietnam, Cao Bang Province | Luong et al. 2023 |
| 71 | *L. phiaoacensis* | IEBR A.5202 | OR405868 | Vietnam, Cao Bang Province | Luong et al. 2023 |
| 72 | *L. phiaoacensis* | IEBR A.5203 | OR405869 | Vietnam, Cao Bang Province | Luong et al. 2023 |
| 73 | *L. phiaoacensis* | IEBR A.5204 | OR405870 | Vietnam, Cao Bang Province | Luong et al. 2023 |
| 74 | *L. phiaoacensis* | IEBR A.5195 | OR405871 | Vietnam, Cao Bang Province | Luong et al. 2023 |
| 75 | *L. pluvialis* | MNHN:1999.5675 | JN848391 | Vietnam, Lao Cai Province | Ohler et al. 2011 |
| 76 | *L. pluvialis* | MNHN:1999.5674 | JN848389 | Vietnam, Lao Cai Province | Ohler et al. 2011 |
| 77 | *L. pluvialis* | ROM_30685 | MH055843 | Vietnam, Lao Cai Province | Chen et al. 2018 |
| 78 | *L. pluvialis* | VNMN 010918 | MZ208923 | Vietnam, Lao Cai Province | Nguyen et al. 2021 |
| 79 | *L. pluvialis* | AMS R186807 | MT644610 | Vietnam, Lao Cai Province | Stuart et al. 2020 |
| 80 | *L. puhoatensis* | AMSR184852 | KY849588 | Vietnam, Nghe An Province | Rowley et al. 2011 |
| 81 | *L. purpurus* | KFY515 | MG520355 | China, Yunnan Province | Yang et al. 2018 |
| 82 | *L. purpuraventra* | SYS:a007306 | MK414531 | China, Guizhou Province | Wang et al. 2019 |
| 83 | *L. pyrrhops* | ZMMU A-5208 | KP017578 | Vietnam, Lam Dong Province | Poyarkov et al. 2015 |
| 84 | *L. rowleyae* | ITBCZ2783 | MG682552 | Vietnam, Da Nang City | Nguyen et al. 2018 |
| 85 | *L. sabahmontana* | BORNEENSIS 12632 | AB847551 | Malaysia, Borneo | Matsui et al. 2014 |
| 86 | *L. sinorensis* | KUHE:19809 | LC741034 | Thailand:Mae Hong Son | Matsui et al. 2023 |
| 87 | *L. shangsiensis* | NHMG<CHN>:1401032 | MK095460 | China, Guangxi Province | Chen et al. 2019 |
| 88 | *L. shimentaina* | SYSa004712 | MH055926 | China, Guangdong Province | Chen et al. 2018 |
| 89 | *L. shiwandashanensis* | NNU202103146 | MZ326691 | China, Guangxi Province | Chen et al. 2021b |
| 90 | *L. sola* | KU_RMB20973 | MH055973 | Malaysia | Chen et al. 2018 |
| 91 | *L. suiyangensis* | GZNU20180606004D3 | MK829650 | China, Guizhou Province | Lou 2020 |
| 92 | *L. sungi* | ZMMU-NAP-06580 | MH055863 | Vietnam, Phu Tho Province | Chen et al. 2018 |
| 93 | *L. tadungensis* | UNS00515 | KR018121 | Vietnam, Dak Nong Province | Rowley et al. 2015 |
| 94 | *L. tengchongensis* | SYSa003766 | MH055897 | China, Yunnan Province | Chen et al. 2018 |
| 95 | *L. tuberosa* | ZMMU-NAP-02275 | MH055959 | Vietnam, Gia Lai Province | Chen et al. 20118 |
| 96 | *L. ventripunctata* | MNHN 2005.0116 | LC201978 | Laos, Phongsaly Province | Matsui et al. 2017 |
| 97 | *L. ventripunctata* | IEBR A.5219 | OR405875 | Vietnam, Cao Bang Province | Luong et al. 2023 |
| 98 | *L. ventripunctata* | IEBR A.5220 | OR405876 | Vietnam, Cao Bang Province | Luong et al. 2023 |
| 99 | *L. ventripunctata* | IEBR A.5221 | OR405877 | Vietnam, Cao Bang Province | Luong et al. 2023 |
| 100 | *L. wuhuangmontis* | SYSa003485 | MH605577 | China, Guangxi Province | Wang et al. 2018 |
| 101 | *L. wulingensis* | CSUFT200 | MT530317 | China, Hunan Province | Qian et al. 2020 |
| 102 | *L. yeae* | CIBEMS20190422HLJ2-1 | MT957022 | China, Sichuan Province | Shi et al. 2021 |
| 103 | *L. yingjiangensis* | SYS a006537 | MG520359 | China, Yunnan Province | Yang et al. 2018 |
| 104 | *L. yingjiangensis* | KIZ027521 | MT302642 |  | Chen et al. 2020 |
| 105 | *L. yunkaiensis* | SYS a004663 | MH605584 | China, Guangdong Province | Wang et al. 2018 |
| 106 | *L. yunyanensis* | GZNU20210622001 | OL800364 | China, Chongqing City | Lou 2022 |
| 107 | *L. zhangyapingi* | KIZ07258 | JX069979 | Thailand, Chiangmai Province | Jiang et al. 2013 |
|  | Outgroup | | | |  |
| 108 | *Leptobrachium* cf*. chapaense* | AMS R171623 | KR018126 | Vietnam, Lao Cai Province | Rowley et al. 2015 |
| 109 | *Megophrys truongsonenensis* | AMS R173870 | KY476333 | Vietnam, Kon Tum Province | Rowley et al. 2017 |
