# Supplementary material for: A new species of Leptobrachella Smith 1925 (Anura, Megophryidae) from Lai Chau Province, Vietnam
Source: Biodivers Data J. 2024 Nov 4;12:e136491. doi: 10.3897/BDJ.12.e136491 (PMC11555430; doi:10.3897/BDJ.12.e136491)
Supplement: Supplementary material 2 — Diagnostic characters on morphology [file bdj-12-e136491-s002.docx]

**Suppl. material 2:** Diagnostic characters on morphology

**Author**: Chung Van Hoang, Anh Mai Luong, Truong Quang Nguyen, Tao Thien Nguyen, Hoa Thi Ninh, Linh Hoang Tu Le, Thomas Ziegler, Cuong The Pham

**Data type**: morphological

**Brief description:** Selected diagnostic characters for the species in the genus *Leptobrachella* occurring north of the Isthmus of Kra (modified from Rowley et al. 2017; Yuan et al. 2017; Nguyen et al. 2018; Wang et al. 2018, Liu et al. 2023; Luong et al. 2023). NA: Not availale.

|  | Species | Male SVL (mm) | Female SVL (mm) | Black spots on flanks | Bicolored iris | Distinct dorsolateral markings | Toes webbing | Fringes on toes | Ventral coloration in life | Dorsal skin texture | References |
| --- | --- | --- | --- | --- | --- | --- | --- | --- | --- | --- | --- |
| 1 | *Leptobrachella huynhi* sp. nov. | NR | 37.8–40.2 | Variable | Absent | Variable | Absent | Narrow | Center of belly cream white and outer edges of belly brown with small whitish spots | Skin on entire dorsum shagreened with low, round tubercles with irregular large and small sizes, alternately arranged and scattered entire dorsum of the body | This study |
| 2 | *L. aerea* | 25.1–28.9 | 27.1–38.6 | Absent | Absent | No | Rudimentary | Wide | Near immaculate creamy white, brown specking on margins | Entire dorsum with low, round or laterally compressed tubercles, more frequent and larger on posterodorsal and dorsolateral surfaces | Rowley et al. 2010c |
| 3 | *L. alpina* | 24.0–26.4 | 31.7–32.5 | Present | Absent | Yes | Rudimentary | Wide in males | Creamy-white with dark spots | Relatively smooth, some with small warts | Fei et al. 1990 |
| 4 | *L. applebyi* | 19.6–22.3 | 21.7 | Present | Absent | Yes | Rudimentary | Absent | Dark brownish pink with white speckling | Smooth | Rowley & Cao 2009 |
| 5 | *L. ardens* | 21.3–24.7 | 25.4 | Present | Absent | Yes | Absent | Absent | Dark brownish red with white speckling | Smooth- finely shagreened | Rowley et al. 2016 |
| 6 | *L. aspera* | 22.4 | 25.0–26.4 | Present | Present | Yes | Rudimentary | Narrow | Creamy white with distinct dark spots | Rough with dense conical granules, tubercles, and dermal ridges | Wang et al. 2020 |
| 7 | *L. aurantirosea* | 21.3–24.4 | NR | Present | Present | Yes | Absent | Narrow | Center of belly, throat and chest cream white; all of the chin, thighs, arms, tibiotarsus and outer edges of belly, throat and chest brown with small whitish spots femoral, pectoral and dorsolateral glands cream white | Skin on entire dorsum shagreened with low, round tubercles with irregular sizes, alternately arranged and scattered | Ninh et al. 2024 |
| 8 | *L. bashaensis* | 22.9–25.6 | 27.1 | Present | Present | Yes | Rudimentary | Narrow | Off-white with dark patches | Shagreened with small tubercles | Lyu et al. 2020 |
| 9 | *L. bidoupensis* | 18.5–25.4 | 29.2–29.4 | Present | Present | Yes | Rudimentary | Narrow | Dark brownish red with white speckling | Smooth | Rowley et al. 2011 |
| 10 | *L. bijie* | 29.9–30.4 | NR | Present | NR | Yes | Rudimentary | Narrow | White with distinct nebulous greyish speckling | Shagreened and granular with short longitudinal dermal ridges | Wang et al. 2019 |
| 11 | *L. botsfordi* | 29.1–32.6 | 30.0–31.8 | Absent | Absent | Yes | Rudimentary | Narrow | Reddish brown with white speckling | Shagreened | Rowley et al. 2013 |
| 12 | *L. bourreti* | 28.0–36.2 | 39.5–45.0 | Present | Present | Yes | Rudimentary | Narrow | Creamy white, light yellow and orange in some specimens | Relatively smooth, some with small warts | Dubois 1983; Ohler et al. 2011 |
| 13 | *L. chishuiensis* | 30.8–33.4 | 34.2 | Present | NR | No | Rudimentary | Narrow | White with grey speckling on margins | Shagreened and granular | Li et al. 2020 |
| 14 | *L. crocea* | 22.2–27.3 | NR | Absent | Absent | No | Rudimentary | Absent | Bright orange | Highly tuberculate | Rowley et al. 2010a |
| 15 | *L. damingshanensis* | 33.6–34.4 | NR | Present | Present | No | Rudimentary | Narrow | Creamy white ventral surface with small, creamy white glands on belly, becoming more concentrated near lateral margin | Rough with small, raised tubercles and ridges | Chen et al. 2021 |
| 16 | *L. dong* | 29.2–32.0 | 34.4–43.1 | Present | Present | Yes | Rudimentary | Wide | White with distinct nebulous brown speckling on ventrolateral flanks | Shagreened with fine tubercles | Liu et al. 2023 |
| 17 | *L. dorsospina* | 28.7–30.5 | 32.1–39.8 | Present | Present | No | Rudimentary | Narrow | Greyish white with dark spots and orange pigmentations | Rough with dense conical granules, tubercles, dermal ridges, and conical spines | Wang et al. 2020 |
| 18 | *L. dushanensis* | 31.9–32.9 | NR | Present | Present | No | Rudimentary | Wide | Ventral light colored with several granules and brown spots | Shagreened with fine tiny granules and short ridges | Li et al. 2024 |
| 19 | *L. eos* | 33.1–34.7 | 40.7 | Absent | Present | No | Rudimentary | Wide | Creamy white | Shagreened | Ohler et al. 2011 |
| 20 | *L. feii* | 21.5–22.8 | 25.7 | Present | Present | Yes | Rudimentary | Narrow | Creamy white with black blotches | Shagreened with small tubercles | Chen et al. 2020 |
| 21 | *L. firthi* | 26.4–29.2 | 25.7–36.9 | Absent | Variable | No | Rudimentary | Wide in males | Creamy white | Shagreened with fine tubercles | Rowley et al. 2012 |
| 22 | *L. flaviglandulosa* | 23.0–27.0 | 29.3 | Present | Present | Yes | Rudimentary | Narrow | Belly creamy white with dark brown dusting along the margins | Shagreened with yellowish brown tubercles | Chen et al. 2020 |
| 23 | *L. fuliginosa* | 28.2–30.0 | NR | Present | Present | Yes | Rudimentary | Narrow | White with brown dusting | Nearly smooth, few tubercles | Matsui 2006 |
| 24 | *L. graminicola* | 23.1–24.6 | 28.6–32.9 | Variable | Present | Yes | Rudimentary | Wide | White with very dark blackish brown spots | Smooth, with many tubercles and lacking dermal ridges | Nguyen et al. 2021 |
| 25 | *L. guinanensis* | 30.5–32.5 | 38.7–41.8 | Present | Present | Yes | Rudimentary | Narrow | Ventral surface creamy white without dark brown spots | Dorsal surface shagreened with small, raised tubercles and longitudinal ridge | Chen et al. 2024 |
| 26 | *L. isos* | 23.7–27.9 | 28.6–31.5 | Absent | Present | No | Rudimentary | Wide in males | Creamy white with white dusting on margins | Mostly smooth, females more tuberculate | Rowley et al. 2015 |
| 27 | *L. jinshaensis* | 29.7–31.2 | NR | Present | Present | Yes | Absent | Narrow | Belly cream yellow with purple speckling | Shagreened, some of the granules forming longitudinal short skin ridges | Cheng et al. 2021 |
| 28 | *L. jinyunensis* | 29.1–34.1 | 34.1–34.9 | Present | Present | Yes | Rudimentary | Narrow | Basically floral white with deep grey pigmens all over | Rough, covered with dense small granules and large tubercles | Shi et al. 2023 |
| 29 | *L. kalonensis* | 25.8–30.6 | 28.9–30.6 | Present | Present | Yes | Absent | Absent | Pale brownish pink with white speckling | Smooth | Rowley et al. 2016 |
| 30 | *L. khasiorum* | 24.5–27.3 | 31.2–33.4 | Present | Present | Yes | Rudimentary | Wide | Creamy white | Isolated, scattered tubercles | Das et al. 2010 |
| 31 | *L. korifi* | NT | 22.7 | Present | NT | Yes | Rudimentary | Narrow | Throat, chest, and abdomen dusted with fine brown network; ventral surfaces of legs dusky, dotted with light brown in preservative | Skin nearly smooth, with tubercles and glandular folds above in preservative | Matsui et al. 2023 |
| 32 | *L. lateralis* | 26.9–28.3 | 36.6 | Present | NR | Yes | Rudimentary | Absent | Creamy white | Roughly granular | Anderson 1871; Humtsoe et al. 2008 |
| 33 | *L. laui* | 24.8–26.7 | 28.1 | Present | Absent | Yes | Rudimentary | Wide | Creamy white with dark brown dusting on margins | Round granular tubercles | Sung et al. 2014 |
| 34 | *L. liui* | 23.0–28.7 | 24.5–27.8 | Present | Absent | Yes | Rudimentary | Wide | Creamy white with dark brown spots on chest and margins | Round granular tubercles with glandular folds | Fei et al. 1990; Ohler et al. 2011; Wang et al. 2020; Nguyen et al. 2021 |
| 35 | *L. macrops* | 28.0–29.3 | 30.3 | Present | Present | No | Rudimentary | Absent | Greyish-violet with white speckling | Roughly granular with larger tubercles | Duong et al. 2018 |
| 36 | *L. maculosa* | 24.2–26.6 | 27 | Present | Present | Yes | Absent | Absent | Dark brownish with white speckling | Mostly smooth | Rowley et al. 2016 |
| 37 | *L. mangshanensis* | 22.2–27.8 | 30.2 | Present | Present | Yes | Rudimentary | Narrow | Reddish brown with white speckling | Nearly smooth, scattered tubercles | Hou et al. 2018 |
| 38 | *L. maoershanensis* | 25.2–30.4 | 29.1 | Present | Present | Yes | Rudimentary | Narrow | Creamy white with irregular black spots | Shagreened with small tubercles and longitudinal ridges | Yuan et al. 2017 |
| 39 | *L. melanoleuca* | 26.6–28.8 | 32.7 | Present | Present | Yes | Rudimentary | Absent | Large black spots on a white background | Skin nearly smooth, scattered with small tubercles | Matsui 2006 |
| 40 | *L. melica* | 19.5–22.7 | NR | Present | Absent | Yes | Rudimentary | Absent | White to pale pink with diffuse dark brown blotches and white speckling | Smooth | Rowley et al. 2010b |
| 41 | *L. minima* | 25.7–31.4 | 31.6–37.3 | Present | Present | Yes | Rudimentary | Absent | Creamy white | Smooth | Ohler et al. 2011; Taylor 1962 |
| 42 | *L. murphyi* | 23.2–24.9 | 29.3–32.1 | Present | Present | Yes | Rudimentary | Wide | Creamy-white belly with small black spots on the margin | Shagreened with reddish tubercles and folds | Chen et al. 2021 |
| 43 | *L. nahangensis* | 40.8 | NR | Present | Absent | Yes | Rudimentary | Absent | Creamy white with light specking on throat and chest | Smooth | Lathrop et al. 1998 |
| 44 | *L. namdongensis* | 30.9 | 32.1–35.3 | Present | Present | No | Rudimentary | Absent | Creamy white with brown dusting on margins | Finely tuberculate | Hoang et al. 2019 |
| 45 | *L. neangi* | NR | 35.4–36.3 | Present | Absent | Yes | Rudimentary | Absent | Belly transparent, immaculate purplish grey in life | Dorsal skin with small irregular bumps and ridges | Stuart & Rowley 2020 |
| 46 | *L. niveumontis* | 22.5–23.6 | 28.5–28.7 | Present | Present | Yes | Rudimentary | Narrow | Marble with irregular black speckling | Relatively smooth with small tubercles | Chen et al. 2020 |
| 47 | *L. nokrekensis* | 26.0–33.0 | 34.0–35.0 | Present | Present | Yes | Rudimentary | unknown | Creamy white | Tubercles and longitudinal folds | Mathew & Sen 2010 |
| 48 | *L. nyx* | 26.7–32.6 | 37.0–41.0 | Present | NR | Yes | Rudimentary | Absent | Creamy white with white with brown margins | Round tubercles | Ohler et al. 2011 |
| 49 | *L. oshanensis* | 26.5–30.5 | 28.8–32.6 | Present | Present | Yes | Absent | Absent | Whitish without markings or only small, light grey spots | Smooth with few glandular ridges | Liu, 1950; Fei et al. 2009, 2012; Shi et al. 2021 |
| 50 | *L. pallida* | 24.5–27.7 | NR | Present | Present | Yes | Absent | Absent | Dark brownish red with faint white speckling | Skin on dorsum coarsely shagreened | Rowley et al. 2016 |
| 51 | *L. pelodytoides* | 27.5–32.3 | NR | Present | UnkAbsentwn | Yes | Wide | Narrow | Whitish | Small, smooth warts | Boulenger 1893; Ohler et al. 2011 |
| 52 | *L. petrops* | 23.6–27.6 | 30.3–47.0 | Present | Present | No | Absent | Narrow | Immaculate creamy white | Highly tuberculate | Rowley et al. 2017b |
| 53 | *L. phiadenensis* | NR | 27.6–28.6 | Present | Present | Yes | Rudimentary | Narrow | Creamy white with brown dusting on margins | Rough with tubercles, and dermal ridges | Luong et al. 2023 |
| 54 | *L. phiaoacensis* | 27.8–33.3 | 31.5–41.8 | Present | Present | No | Rudimentary | Narrow | Belly from pinkish white to white with dark brown specking on belly periphery | Shagreened with fine tubercles, denser in posterior part of the back | Luong et al. 2023 |
| 55 | *L. pluvialis* | 21.3–27.52 | 25.5–33.5 | Present | Present | Yes | Absent | Absent | Dirty white with dark brown marbling or dark spots | Smooth, flattened tubercles on flanks | Ohler et al. 2000; Nguyen et al. 2021 |
| 56 | *L. puhoatensis* | 24.2–28.1 | 27.3–31.5 | Present | Present | Yes | Rudimentary | Narrow | Reddish brown with white dusting | Longitudinal skin ridges | Rowley et al. 2017a |
| 57 | *L. purpuraventra* | 27.3–29.8 | 33.0–35.3 | Present | Present | Yes | Rudimentary | Narrow | Greyish white with nebulous dark speckling | Shagreened and granular with dermal ridges | Wang et al. 2019 |
| 58 | *L. purpurus* | 25.7–27.5 | NR | Present | Present | Yes | Rudimentary | Wide | Dull white with indistinct grey dusting to dull grey | Shagreened with small tubercles | Yang et al. 2018 |
| 59 | *L. pyrrhops* | 30.8–34.3 | 30.8–34.3 | Present | Present | Yes | Rudimentary | Absent | Grey pinkish to dark brownish -violet | Slightly shagreened | Poyarkov et al. 2015 |
| 60 | *L.* *rowleyae* | 23.4–25.4 | 27.0–27.8 | Present | Present | No | Rudimentary | Absent | Pinkish milk-white with dense whitish speckling evenly scattered on entire ventral surface | mostly smooth with numerous tiny tubercles and pustules finely | Nguyen et al. 2018 |
| 61 | *L. shangsiensis* | 24.9–29.4 | 30.8–35.9 | Present | Present | No | Rudimentary | Narrow | Yellowish creamy-white with marble texture | Mostly smooth with numerous tiny tubercles | Chen et al. 2019 |
| 62 | *L. shimentaina* | 26.4–28.9 | 30.1–30.7 | Present | Present | Yes | Rudimentary | Wide | Grayish pink with distinct hazy brown speckling | Shagreened and granular | Wang et al. 2022 |
| 63 | *L. shiwandashanensis* | 24.3–29.7 | 32.3–35.9 | Present | Present | No | Absent | Absent | Creamy white with brown spots on lateral margin | Shagreened with small tubercles and ridges | Chen et al. 2021; Lo et al. 2022 |
| 64 | *L. sinorensis* | 26.6–27.1 | NR | Present | Present | Yes | Rudimentary | Narrow | Throat purplish; chest and abdomen milky white; ventral surfaces of legs purplish, dotted with white | Skin nearly smooth, with a few tubercles of varying sizes above | Matsui et al. 2023 |
| 65 | *L. suiyangensis* | 28.7–29.7 | 30.5–33.5 | Present | Present | No | Rudimentary | Narrow | Yellowish creamy-white with brown speckling | Shagreened with small granules | Luo et al. 2020 |
| 66 | *L. sungi* | 48.3–52.7 | 56.7–58.9 | Absent or small | Absent | Yes | Wide | Narrow | White | Granular | Lathrop et al. 1998 |
| 67 | *L. tadungensis* | 23.3–28.2 | 32.1 | Present | Absent | Yes | Absent | Absent | Dark brownish with white speckling | Smooth | Rowley et al. 2016 |
| 68 | *L. tamdil* | 32.3 | 32.3 | Present | Present | Yes | Wide | Wide | White | Weakly tuberculate | Sengupta et al. 2010 |
| 69 | *L. tengchongensis* | 23.9–26.0 | 28.8–28.9 | Present | Absent | Yes | Rudimentary | Narrow | White with dark brown speckling | Shagreened with small tubercles | Yang et al. 2016 |
| 70 | *L. tuberosa* | 24.4–29.5 | 30.2 | Absent | Absent | No | Rudimentary | Absent | White with small grey spots/streaks | Highly tuberculate | Inger et al. 1999; Rowley et al. 2010a |
| 71 | *L. ventripunctata* | 25.5–28.0 | 31.5–35.0 | Present | Present | Yes | Rudimentary | Absent | Chest and belly creamy white with dark brown spots | Longitudinal skin ridges | Fei et al. 1991, 2009, 2012 |
| 72 | *L. verrucosa* | 23.2–25.9 | NR | Absent | Present | Yes | Rudimentary | Narrow | Creamy white with distinct grayish white and dark brown spots | Shagreened with numerous conical tubercles | Lin et al. 2022 |
| 73 | *L. wuhuangmontis* | 25.6–30.0 | 33.0–36.0 | Present | Present | Yes | Rudimentary | Narrow | Greyish white mixed by tiny white and black dots | Rough, scattered with dense conical tubercles | Wang et al. 2018 |
| 74 | *L. wulingensis* | 24.5–32.8 | 29.9–38.5 | Present | Present | Yes | Rudimentary | Narrow | Creamy white but translucent with greyish-pink skin color | Shagreened with sparse large warts, sometimes with longitudinal dermal ridges | Qian et al. 2020 |
| 75 | *L. wumingensis* | 26-26.7 | 30.6-34.8 | Present | Present | No | Absent | Absent | Belly immaculate creamy white, throat and chest creamy with tiny light brown spots | Shagreened and granular | Chen et al. 2023 |
| 76 | *L. yeae* | 25.8–31.2 | 33.7–34.1 | Present | Present | Yes | Rudimentary | Narrow | Cream white with brown specking on sides and upper abdomen | Relatively smooth with tiny granules | Shi et al. 2021 |
| 77 | *L. yingjiangensis* | 25.7–27.6 | NR | Present | Present | Yes | Rudimentary | Wide | Creamy white with dark brown flecks on margin | Shagreened with small tubercles | Yang et al. 2018 |
| 78 | *L. yunkaiensis* | 25.9–29.3 | 34.0–35.3 | Present | Present | No | Rudimentary | Wide | Belly pinkish with distinct or indistinct speckling | Shagreened with fine tubercles and short skin ridges | Wang et al. 2018 |
| 79 | *L. yunyangensis* | 28.3–30.6 | NR | Present | Present | Yes | Rudimentary | Narrow | Greyish white with purple-brown speckling | Skin rough, with sparse large granules and tubercles | Luo et al. 2022 |
| 80 | *L. zhangyapingi* | 45.8–52.5 | NR | Absent | Present | Narrow | Rudimentary | Wide | Creamy-white with white with brown margins | Mostly smooth with distinct tubercles | Jiang et al. 2013 |

|  | Species | Male SVL (mm) | Female SVL (mm) | Black spots on flanks | Bicolored iris | Distinct dorsolateral markings | Toes webbing | Fringes on toes | Ventral coloration in life | Dorsal skin texture | References |
| --- | --- | --- | --- | --- | --- | --- | --- | --- | --- | --- | --- |
| 1 | *Leptobrachella huynhi* sp. nov. | NR | 37.8–40.2 | Variable | Absent | Variable | Absent | Narrow | Center of belly cream white and outer edges of belly brown with small whitish spots | Skin on entire dorsum shagreened with low, round tubercles with irregular large and small sizes, alternately arranged and scattered entire dorsum of the body | This study |
| 2 | *L. aerea* | 25.1–28.9 | 27.1–38.6 | Absent | Absent | No | Rudimentary | Wide | Near immaculate creamy white, brown specking on margins | Entire dorsum with low, round or laterally compressed tubercles, more frequent and larger on posterodorsal and dorsolateral surfaces | Rowley et al. 2010c |
| 3 | *L. alpina* | 24.0–26.4 | 31.7–32.5 | Present | Absent | Yes | Rudimentary | Wide in males | Creamy-white with dark spots | Relatively smooth, some with small warts | Fei et al. 1990 |
| 4 | *L. applebyi* | 19.6–22.3 | 21.7 | Present | Absent | Yes | Rudimentary | Absent | Dark brownish pink with white speckling | Smooth | Rowley & Cao 2009 |
| 5 | *L. ardens* | 21.3–24.7 | 25.4 | Present | Absent | Yes | Absent | Absent | Dark brownish red with white speckling | Smooth- finely shagreened | Rowley et al. 2016 |
| 6 | *L. aspera* | 22.4 | 25.0–26.4 | Present | Present | Yes | Rudimentary | Narrow | Creamy white with distinct dark spots | Rough with dense conical granules, tubercles, and dermal ridges | Wang et al. 2020 |
| 7 | *L. aurantirosea* | 21.3–24.4 | NR | Present | Present | Yes | Absent | Narrow | Center of belly, throat and chest cream white; all of the chin, thighs, arms, tibiotarsus and outer edges of belly, throat and chest brown with small whitish spots femoral, pectoral and dorsolateral glands cream white | Skin on entire dorsum shagreened with low, round tubercles with irregular sizes, alternately arranged and scattered | Ninh et al. 2024 |
| 8 | *L. bashaensis* | 22.9–25.6 | 27.1 | Present | Present | Yes | Rudimentary | Narrow | Off-white with dark patches | Shagreened with small tubercles | Lyu et al. 2020 |
| 9 | *L. bidoupensis* | 18.5–25.4 | 29.2–29.4 | Present | Present | Yes | Rudimentary | Narrow | Dark brownish red with white speckling | Smooth | Rowley et al. 2011 |
| 10 | *L. bijie* | 29.9–30.4 | NR | Present | NR | Yes | Rudimentary | Narrow | White with distinct nebulous greyish speckling | Shagreened and granular with short longitudinal dermal ridges | Wang et al. 2019 |
| 11 | *L. botsfordi* | 29.1–32.6 | 30.0–31.8 | Absent | Absent | Yes | Rudimentary | Narrow | Reddish brown with white speckling | Shagreened | Rowley et al. 2013 |
| 12 | *L. bourreti* | 28.0–36.2 | 39.5–45.0 | Present | Present | Yes | Rudimentary | Narrow | Creamy white, light yellow and orange in some specimens | Relatively smooth, some with small warts | Dubois 1983; Ohler et al. 2011 |
| 13 | *L. chishuiensis* | 30.8–33.4 | 34.2 | Present | NR | No | Rudimentary | Narrow | White with grey speckling on margins | Shagreened and granular | Li et al. 2020 |
| 14 | *L. crocea* | 22.2–27.3 | NR | Absent | Absent | No | Rudimentary | Absent | Bright orange | Highly tuberculate | Rowley et al. 2010a |
| 15 | *L. damingshanensis* | 33.6–34.4 | NR | Present | Present | No | Rudimentary | Narrow | Creamy white ventral surface with small, creamy white glands on belly, becoming more concentrated near lateral margin | Rough with small, raised tubercles and ridges | Chen et al. 2021 |
| 16 | *L. dong* | 29.2–32.0 | 34.4–43.1 | Present | Present | Yes | Rudimentary | Wide | White with distinct nebulous brown speckling on ventrolateral flanks | Shagreened with fine tubercles | Liu et al. 2023 |
| 17 | *L. dorsospina* | 28.7–30.5 | 32.1–39.8 | Present | Present | No | Rudimentary | Narrow | Greyish white with dark spots and orange pigmentations | Rough with dense conical granules, tubercles, dermal ridges, and conical spines | Wang et al. 2020 |
| 18 | *L. dushanensis* | 31.9–32.9 | NR | Present | Present | No | Rudimentary | Wide | Ventral light colored with several granules and brown spots | Shagreened with fine tiny granules and short ridges | Li et al. 2024 |
| 19 | *L. eos* | 33.1–34.7 | 40.7 | Absent | Present | No | Rudimentary | Wide | Creamy white | Shagreened | Ohler et al. 2011 |
| 20 | *L. feii* | 21.5–22.8 | 25.7 | Present | Present | Yes | Rudimentary | Narrow | Creamy white with black blotches | Shagreened with small tubercles | Chen et al. 2020 |
| 21 | *L. firthi* | 26.4–29.2 | 25.7–36.9 | Absent | Variable | No | Rudimentary | Wide in males | Creamy white | Shagreened with fine tubercles | Rowley et al. 2012 |
| 22 | *L. flaviglandulosa* | 23.0–27.0 | 29.3 | Present | Present | Yes | Rudimentary | Narrow | Belly creamy white with dark brown dusting along the margins | Shagreened with yellowish brown tubercles | Chen et al. 2020 |
| 23 | *L. fuliginosa* | 28.2–30.0 | NR | Present | Present | Yes | Rudimentary | Narrow | White with brown dusting | Nearly smooth, few tubercles | Matsui 2006 |
| 24 | *L. graminicola* | 23.1–24.6 | 28.6–32.9 | Variable | Present | Yes | Rudimentary | Wide | White with very dark blackish brown spots | Smooth, with many tubercles and lacking dermal ridges | Nguyen et al. 2021 |
| 25 | *L. guinanensis* | 30.5–32.5 | 38.7–41.8 | Present | Present | Yes | Rudimentary | Narrow | Ventral surface creamy white without dark brown spots | Dorsal surface shagreened with small, raised tubercles and longitudinal ridge | Chen et al. 2024 |
| 26 | *L. isos* | 23.7–27.9 | 28.6–31.5 | Absent | Present | No | Rudimentary | Wide in males | Creamy white with white dusting on margins | Mostly smooth, females more tuberculate | Rowley et al. 2015 |
| 27 | *L. jinshaensis* | 29.7–31.2 | NR | Present | Present | Yes | Absent | Narrow | Belly cream yellow with purple speckling | Shagreened, some of the granules forming longitudinal short skin ridges | Cheng et al. 2021 |
| 28 | *L. jinyunensis* | 29.1–34.1 | 34.1–34.9 | Present | Present | Yes | Rudimentary | Narrow | Basically floral white with deep grey pigmens all over | Rough, covered with dense small granules and large tubercles | Shi et al. 2023 |
| 29 | *L. kalonensis* | 25.8–30.6 | 28.9–30.6 | Present | Present | Yes | Absent | Absent | Pale brownish pink with white speckling | Smooth | Rowley et al. 2016 |
| 30 | *L. khasiorum* | 24.5–27.3 | 31.2–33.4 | Present | Present | Yes | Rudimentary | Wide | Creamy white | Isolated, scattered tubercles | Das et al. 2010 |
| 31 | *L. korifi* | NT | 22.7 | Present | NT | Yes | Rudimentary | Narrow | Throat, chest, and abdomen dusted with fine brown network; ventral surfaces of legs dusky, dotted with light brown in preservative | Skin nearly smooth, with tubercles and glandular folds above in preservative | Matsui et al. 2023 |
| 32 | *L. lateralis* | 26.9–28.3 | 36.6 | Present | NR | Yes | Rudimentary | Absent | Creamy white | Roughly granular | Anderson 1871; Humtsoe et al. 2008 |
| 33 | *L. laui* | 24.8–26.7 | 28.1 | Present | Absent | Yes | Rudimentary | Wide | Creamy white with dark brown dusting on margins | Round granular tubercles | Sung et al. 2014 |
| 34 | *L. liui* | 23.0–28.7 | 24.5–27.8 | Present | Absent | Yes | Rudimentary | Wide | Creamy white with dark brown spots on chest and margins | Round granular tubercles with glandular folds | Fei et al. 1990; Ohler et al. 2011; Wang et al. 2020; Nguyen et al. 2021 |
| 35 | *L. macrops* | 28.0–29.3 | 30.3 | Present | Present | No | Rudimentary | Absent | Greyish-violet with white speckling | Roughly granular with larger tubercles | Duong et al. 2018 |
| 36 | *L. maculosa* | 24.2–26.6 | 27 | Present | Present | Yes | Absent | Absent | Dark brownish with white speckling | Mostly smooth | Rowley et al. 2016 |
| 37 | *L. mangshanensis* | 22.2–27.8 | 30.2 | Present | Present | Yes | Rudimentary | Narrow | Reddish brown with white speckling | Nearly smooth, scattered tubercles | Hou et al. 2018 |
| 38 | *L. maoershanensis* | 25.2–30.4 | 29.1 | Present | Present | Yes | Rudimentary | Narrow | Creamy white with irregular black spots | Shagreened with small tubercles and longitudinal ridges | Yuan et al. 2017 |
| 39 | *L. melanoleuca* | 26.6–28.8 | 32.7 | Present | Present | Yes | Rudimentary | Absent | Large black spots on a white background | Skin nearly smooth, scattered with small tubercles | Matsui 2006 |
| 40 | *L. melica* | 19.5–22.7 | NR | Present | Absent | Yes | Rudimentary | Absent | White to pale pink with diffuse dark brown blotches and white speckling | Smooth | Rowley et al. 2010b |
| 41 | *L. minima* | 25.7–31.4 | 31.6–37.3 | Present | Present | Yes | Rudimentary | Absent | Creamy white | Smooth | Ohler et al. 2011; Taylor 1962 |
| 42 | *L. murphyi* | 23.2–24.9 | 29.3–32.1 | Present | Present | Yes | Rudimentary | Wide | Creamy-white belly with small black spots on the margin | Shagreened with reddish tubercles and folds | Chen et al. 2021 |
| 43 | *L. nahangensis* | 40.8 | NR | Present | Absent | Yes | Rudimentary | Absent | Creamy white with light specking on throat and chest | Smooth | Lathrop et al. 1998 |
| 44 | *L. namdongensis* | 30.9 | 32.1–35.3 | Present | Present | No | Rudimentary | Absent | Creamy white with brown dusting on margins | Finely tuberculate | Hoang et al. 2019 |
| 45 | *L. neangi* | NR | 35.4–36.3 | Present | Absent | Yes | Rudimentary | Absent | Belly transparent, immaculate purplish grey in life | Dorsal skin with small irregular bumps and ridges | Stuart & Rowley 2020 |
| 46 | *L. niveumontis* | 22.5–23.6 | 28.5–28.7 | Present | Present | Yes | Rudimentary | Narrow | Marble with irregular black speckling | Relatively smooth with small tubercles | Chen et al. 2020 |
| 47 | *L. nokrekensis* | 26.0–33.0 | 34.0–35.0 | Present | Present | Yes | Rudimentary | unknown | Creamy white | Tubercles and longitudinal folds | Mathew & Sen 2010 |
| 48 | *L. nyx* | 26.7–32.6 | 37.0–41.0 | Present | NR | Yes | Rudimentary | Absent | Creamy white with white with brown margins | Round tubercles | Ohler et al. 2011 |
| 49 | *L. oshanensis* | 26.5–30.5 | 28.8–32.6 | Present | Present | Yes | Absent | Absent | Whitish without markings or only small, light grey spots | Smooth with few glandular ridges | Liu, 1950; Fei et al. 2009, 2012; Shi et al. 2021 |
| 50 | *L. pallida* | 24.5–27.7 | NR | Present | Present | Yes | Absent | Absent | Dark brownish red with faint white speckling | Skin on dorsum coarsely shagreened | Rowley et al. 2016 |
| 51 | *L. pelodytoides* | 27.5–32.3 | NR | Present | UnkAbsentwn | Yes | Wide | Narrow | Whitish | Small, smooth warts | Boulenger 1893; Ohler et al. 2011 |
| 52 | *L. petrops* | 23.6–27.6 | 30.3–47.0 | Present | Present | No | Absent | Narrow | Immaculate creamy white | Highly tuberculate | Rowley et al. 2017b |
| 53 | *L. phiadenensis* | NR | 27.6–28.6 | Present | Present | Yes | Rudimentary | Narrow | Creamy white with brown dusting on margins | Rough with tubercles, and dermal ridges | Luong et al. 2023 |
| 54 | *L. phiaoacensis* | 27.8–33.3 | 31.5–41.8 | Present | Present | No | Rudimentary | Narrow | Belly from pinkish white to white with dark brown specking on belly periphery | Shagreened with fine tubercles, denser in posterior part of the back | Luong et al. 2023 |
| 55 | *L. pluvialis* | 21.3–27.52 | 25.5–33.5 | Present | Present | Yes | Absent | Absent | Dirty white with dark brown marbling or dark spots | Smooth, flattened tubercles on flanks | Ohler et al. 2000; Nguyen et al. 2021 |
| 56 | *L. puhoatensis* | 24.2–28.1 | 27.3–31.5 | Present | Present | Yes | Rudimentary | Narrow | Reddish brown with white dusting | Longitudinal skin ridges | Rowley et al. 2017a |
| 57 | *L. purpuraventra* | 27.3–29.8 | 33.0–35.3 | Present | Present | Yes | Rudimentary | Narrow | Greyish white with nebulous dark speckling | Shagreened and granular with dermal ridges | Wang et al. 2019 |
| 58 | *L. purpurus* | 25.7–27.5 | NR | Present | Present | Yes | Rudimentary | Wide | Dull white with indistinct grey dusting to dull grey | Shagreened with small tubercles | Yang et al. 2018 |
| 59 | *L. pyrrhops* | 30.8–34.3 | 30.8–34.3 | Present | Present | Yes | Rudimentary | Absent | Grey pinkish to dark brownish -violet | Slightly shagreened | Poyarkov et al. 2015 |
| 60 | *L.* *rowleyae* | 23.4–25.4 | 27.0–27.8 | Present | Present | No | Rudimentary | Absent | Pinkish milk-white with dense whitish speckling evenly scattered on entire ventral surface | mostly smooth with numerous tiny tubercles and pustules finely | Nguyen et al. 2018 |
| 61 | *L. shangsiensis* | 24.9–29.4 | 30.8–35.9 | Present | Present | No | Rudimentary | Narrow | Yellowish creamy-white with marble texture | Mostly smooth with numerous tiny tubercles | Chen et al. 2019 |
| 62 | *L. shimentaina* | 26.4–28.9 | 30.1–30.7 | Present | Present | Yes | Rudimentary | Wide | Grayish pink with distinct hazy brown speckling | Shagreened and granular | Wang et al. 2022 |
| 63 | *L. shiwandashanensis* | 24.3–29.7 | 32.3–35.9 | Present | Present | No | Absent | Absent | Creamy white with brown spots on lateral margin | Shagreened with small tubercles and ridges | Chen et al. 2021; Lo et al. 2022 |
| 64 | *L. sinorensis* | 26.6–27.1 | NR | Present | Present | Yes | Rudimentary | Narrow | Throat purplish; chest and abdomen milky white; ventral surfaces of legs purplish, dotted with white | Skin nearly smooth, with a few tubercles of varying sizes above | Matsui et al. 2023 |
| 65 | *L. suiyangensis* | 28.7–29.7 | 30.5–33.5 | Present | Present | No | Rudimentary | Narrow | Yellowish creamy-white with brown speckling | Shagreened with small granules | Luo et al. 2020 |
| 66 | *L. sungi* | 48.3–52.7 | 56.7–58.9 | Absent or small | Absent | Yes | Wide | Narrow | White | Granular | Lathrop et al. 1998 |
| 67 | *L. tadungensis* | 23.3–28.2 | 32.1 | Present | Absent | Yes | Absent | Absent | Dark brownish with white speckling | Smooth | Rowley et al. 2016 |
| 68 | *L. tamdil* | 32.3 | 32.3 | Present | Present | Yes | Wide | Wide | White | Weakly tuberculate | Sengupta et al. 2010 |
| 69 | *L. tengchongensis* | 23.9–26.0 | 28.8–28.9 | Present | Absent | Yes | Rudimentary | Narrow | White with dark brown speckling | Shagreened with small tubercles | Yang et al. 2016 |
| 70 | *L. tuberosa* | 24.4–29.5 | 30.2 | Absent | Absent | No | Rudimentary | Absent | White with small grey spots/streaks | Highly tuberculate | Inger et al. 1999; Rowley et al. 2010a |
| 71 | *L. ventripunctata* | 25.5–28.0 | 31.5–35.0 | Present | Present | Yes | Rudimentary | Absent | Chest and belly creamy white with dark brown spots | Longitudinal skin ridges | Fei et al. 1991, 2009, 2012 |
| 72 | *L. verrucosa* | 23.2–25.9 | NR | Absent | Present | Yes | Rudimentary | Narrow | Creamy white with distinct grayish white and dark brown spots | Shagreened with numerous conical tubercles | Lin et al. 2022 |
| 73 | *L. wuhuangmontis* | 25.6–30.0 | 33.0–36.0 | Present | Present | Yes | Rudimentary | Narrow | Greyish white mixed by tiny white and black dots | Rough, scattered with dense conical tubercles | Wang et al. 2018 |
| 74 | *L. wulingensis* | 24.5–32.8 | 29.9–38.5 | Present | Present | Yes | Rudimentary | Narrow | Creamy white but translucent with greyish-pink skin color | Shagreened with sparse large warts, sometimes with longitudinal dermal ridges | Qian et al. 2020 |
| 75 | *L. wumingensis* | 26-26.7 | 30.6-34.8 | Present | Present | No | Absent | Absent | Belly immaculate creamy white, throat and chest creamy with tiny light brown spots | Shagreened and granular | Chen et al. 2023 |
| 76 | *L. yeae* | 25.8–31.2 | 33.7–34.1 | Present | Present | Yes | Rudimentary | Narrow | Cream white with brown specking on sides and upper abdomen | Relatively smooth with tiny granules | Shi et al. 2021 |
| 77 | *L. yingjiangensis* | 25.7–27.6 | NR | Present | Present | Yes | Rudimentary | Wide | Creamy white with dark brown flecks on margin | Shagreened with small tubercles | Yang et al. 2018 |
| 78 | *L. yunkaiensis* | 25.9–29.3 | 34.0–35.3 | Present | Present | No | Rudimentary | Wide | Belly pinkish with distinct or indistinct speckling | Shagreened with fine tubercles and short skin ridges | Wang et al. 2018 |
| 79 | *L. yunyangensis* | 28.3–30.6 | NR | Present | Present | Yes | Rudimentary | Narrow | Greyish white with purple-brown speckling | Skin rough, with sparse large granules and tubercles | Luo et al. 2022 |
| 80 | *L. zhangyapingi* | 45.8–52.5 | NR | Absent | Present | Narrow | Rudimentary | Wide | Creamy-white with white with brown margins | Mostly smooth with distinct tubercles | Jiang et al. 2013 |
