# Supplementary material for: A new species of Leptobrachella Smith 1925 (Anura, Megophryidae) from Lai Chau Province, Vietnam
Source: Biodivers Data J. 2024 Nov 4;12:e136491. doi: 10.3897/BDJ.12.e136491 (PMC11555430; doi:10.3897/BDJ.12.e136491)
Supplement: Supplementary material 3 — Uncorrected (“p”) distance matrix [file bdj-12-e136491-s003.docx]

**Suppl. material 3:** Uncorrected (“p”) distance matrix showing percentage pair wise genetic divergence (16S gene) between analysed members of the *Leptobrachella* species

**Author**: Chung Van Hoang, Anh Mai Luong, Truong Quang Nguyen, Tao Thien Nguyen, Hoa Thi Ninh, Linh Hoang Tu Le, Thomas Ziegler, Cuong The Pham

**Data type**: phylogenetic

**Brief description**: Uncorrected (“p”) distance matrix showing percentage pair wise genetic divergence (16S gene) between analysed members of the *Leptobrachella* species

|  | **Species** | **1** | **2** | **3** | **4** | **5** | **6** | **7** | **8** | **9** | **10** | **11** | **12** | **13** | **14** | **15** |
| --- | --- | --- | --- | --- | --- | --- | --- | --- | --- | --- | --- | --- | --- | --- | --- | --- |
| **1** | *Leptobrachella huynhi* sp. nov. | **0.00** |  |  |  |  |  |  |  |  |  |  |  |  |  |  |
| **2** | *L. pluvialis* | 4.38–5.29 | **0.00** |  |  |  |  |  |  |  |  |  |  |  |  |  |
| **3** | *L. minima* | 6.71 | 8.09–9.77 | **0.00** |  |  |  |  |  |  |  |  |  |  |  |  |
| **4** | *L. nyx* | 3.62 | 5.75–6.71 | 5.91–7.22 | **0.00–1.31** |  |  |  |  |  |  |  |  |  |  |  |
| **5** | *L. ventripunctata* | 6.01–6.22 | 6.40–9.15 | 7.84–7.86 | 5.85–6.26 | **0.00–1.68** |  |  |  |  |  |  |  |  |  |  |
| **6** | *L. nahangensis* | 6.17 | 6.39–7.39 | 7.11 | 3.80–4.87 | 7.07–7.54 | **0.00** |  |  |  |  |  |  |  |  |  |
| **7** | *L. aerea* | 6.12 | 6.22–7.94 | 5.91 | 4.85–5.72 | 6.12–6.57 | 6.13 | **0.00** |  |  |  |  |  |  |  |  |
| **8** | *L. daminhshanensis* | 7.60 | 8.07–10.00 | 8.29 | 5.31–6.14 | 7.19 | 6.60 | 7.86 | **0.00** |  |  |  |  |  |  |  |
| **9** | *L. aspera* | 6.16 | 7.59–9.33 | 8.20 | 6.88–7.32 | 7.53–7.75 | 8.62 | 5.70 | 9.06 | **0.00** |  |  |  |  |  |  |
| **10** | *L. sungi* | 7.29 | 8.21–9.95 | 9.64 | 7.57–7.80 | 8.67–9.58 | 8.87 | 8.35 | 10.49 | 9.61 | **0.00** |  |  |  |  |  |
| **11** | *L. shiwandashanensis* | 8.33 | 9.72–11.84 | 10.17 | 8.86–9.29 | 10.56–11.28 | 8.97 | 8.24 | 10.96 | 8.71 | 11.56 | **0.00** |  |  |  |  |
| **12** | *L. wuhuangmontis* | 8.02 | 9.22–10.96 | 10.43 | 7.16–7.36 | 9.44–9.91 | 8.33 | 8.52 | 10.83 | 10.21 | 11.14 | 7.79 | **0.00** |  |  |  |
| **13** | *L. bashaensis* | 8.57 | 8.15–9.84 | 9.24 | 8.21–8.42 | 10.73–11.19 | 8.53 | 8.25 | 9.87 | 8.48 | 10.55 | 9.53 | 9.79 | **0.00** |  |  |
| **14** | *L. dong* | 9.47 | 9.54–11.79 | 10.67 | 8.55–8.77 | 10.89–11.63 | 9.51 | 9.43 | 11.82 | 10.15 | 9.75 | 12.19 | 10.74 | 8.33 | **0.00** |  |
| **15** | *L. feii* | 6.13 | 7.82–9.92 | 7.93 | 6.40–6.84 | 7.27–7.48 | 8.36 | 5.01 | 8.33 | 3.11 | 10.04 | 8.69 | 9.92 | 9.20 | 10.90 | **0.00** |
| **16** | *L. flaviglandulosa* | 7.42 | 6.98–8.16 | 9.57 | 6.33–7.01 | 8.63–9.57 | 7.90 | 7.64 | 9.96 | 9.46 | 9.70 | 11.72 | 10.79 | 5.94 | 8.40 | 9.73 |
| **17** | *L. phiaoacensis* | 8.87–9.06 | 8.47–9.72 | 9.10–9.79 | 8.35–9.56 | 9.70–11.10 | 9.08–9.29 | 8.81–9.05 | 10.70–11.12 | 9.97–10.41 | 10.48–10.69 | 12.86–12.89 | 11.52–11.97 | 6.59–7.17 | 9.10–9.81 | 10.95–11.39 |
| **18** | *L. phiadenensis* | 7.46 | 6.86–7.28 | 9.25 | 6.69–7.09 | 8.76–9.62 | 7.10 | 7.97 | 9.43 | 8.69 | 10.05 | 8.57 | 9.21 | 8.79 | 11.63 | 8.92 |
| **19** | *L. jinyunensis* | 12.20 | 11.63–14.29 | 13.75 | 11.48–11.95 | 12.48–12.71 | 11.57 | 11.46 | 14.33 | 13.53 | 14.36 | 15.34 | 12.82 | 11.22 | 5.08 | 13.03 |
| **20** | *L. korifi* | 13.88 | 14.49–16.45 | 15.92 | 13.92–14.38 | 14.28–15.06 | 13.87 | 15.64 | 14.59 | 14.01 | 14.12 | 16.93 | 17.93 | 12.85 | 10.58 | 15.39 |
| **21** | *L. sinorensis* | 13.49 | 14.08–16.54 | 15.35 | 13.60–13.83 | 15.28–17.74 | 14.07 | 13.47 | 16.16 | 13.75 | 14.28 | 16.14 | 15.44 | 12.17 | 9.50 | 14.59 |
| **22** | *L. yunyanensis* | 11.72 | 10.80–14.03 | 12.27 | 11.09–11.52 | 12.42–12.46 | 11.67 | 9.70 | 13.44 | 10.26 | 10.71 | 14.16 | 12.72 | 10.62 | 5.31 | 10.74 |
| **23** | *L. murphyi* | 12.80 | 12.62–15.48 | 12.98 | 12.80–13.28 | 13.52–13.78 | 12.05 | 13.88 | 14.69 | 13.24 | 13.77 | 14.37 | 14.38 | 12.48 | 8.53 | 13.76 |
| **24** | *L. oshanensis* | 10.45 | 10.35–13.44 | 11.64 | 10.46 | 11.15–11.17 | 9.77 | 9.71 | 12.70 | 9.99 | 10.93 | 13.40 | 12.24 | 10.39 | 6.61 | 10.74 |
| **25** | *L. graminicola* | 11.46–12.17 | 11.27–13.81 | 12.94–13.89 | 10.17–10.76 | 12.83–14.28 | 9.01–9.52 | 10.88–11.60 | 13.51–14.22 | 11.19–12.24 | 11.74–12.01 | 13.76–13.96 | 11.81–11.88 | 9.69–10.23 | 2.41–3.06 | 12.74–13.69 |
| **26** | *L. dorsospina* | 10.96 | 10.81–13.16 | 10.19 | 9.80 | 11.50–12.22 | 10.54 | 10.18 | 11.87 | 10.91 | 11.70 | 13.23 | 11.78 | 9.04 | 3.98 | 12.15 |
| **27** | *L. yeae* | 10.63 | 10.49–13.29 | 11.12 | 9.73–10.61 | 11.10–12.01 | 9.52 | 10.13 | 12.22 | 10.63 | 10.90 | 13.97 | 11.67 | 9.47 | 3.33 | 11.86 |
| **28** | *L. jinshaensis* | 10.42 | 10.27–12.45 | 11.61 | 9.49–9.94 | 11.15–11.16 | 10.68 | 9.90 | 12.28 | 10.62 | 11.87 | 13.38 | 11.69 | 9.92 | 3.11 | 10.89 |
| **29** | *L. neangi* | 11.49 | 12.79–16.19 | 15.04 | 11.24–11.90 | 13.96–14.65 | 11.18 | 12.48 | 12.73 | 11.68 | 13.40 | 14.16 | 12.44 | 12.50 | 13.41 | 12.87 |
| **30** | *L. wulingensis* | 9.65 | 10.08–12.90 | 11.56 | 9.41–9.64 | 10.84–11.55 | 9.54 | 10.65 | 11.69 | 9.27 | 11.62 | 13.06 | 11.22 | 8.44 | 3.98 | 10.94 |
| **31** | *L. yingjiangensis* | 10.36–11.69 | 10.59–13.28 | 10.89–11.82 | 10.48–12.28 | 12.43–14.23 | 10.66 | 11.58 | 13.88–14.45 | 12.10 | 12.81 | 13.10–13.73 | 13.41–13.44 | 9.35–10.47 | 9.70 | 13.15 |
| **32** | *L. niveimontis* | 11.08 | 10.48–12.74 | 11.41 | 8.75–9.20 | 10.91–11.62 | 10.65 | 10.33 | 12.02 | 10.41 | 11.16 | 13.90 | 12.61 | 9.28 | 4.20 | 11.33 |
| **33** | *L. chishuiensis* | 10.02 | 9.87–12.26 | 11.64 | 9.78–10.24 | 11.45–11.47 | 10.01 | 9.92 | 11.83 | 11.11 | 12.88 | 13.21 | 11.95 | 9.95 | 4.41 | 11.38 |
| **34** | *L. suiyangensis* | 10.95 | 10.47–13.55 | 13.45 | 10.74–11.17 | 12.53–12.72 | 11.61 | 10.81 | 13.56 | 11.08 | 12.10 | 14.03 | 13.12 | 11.59 | 3.99 | 10.65 |
| **35** | *L. bijie* | 12.66 | 11.22–14.56 | 13.24 | 12.20–12.86 | 13.17–13.37 | 11.40 | 10.61 | 13.57 | 12.55 | 13.09 | 14.05 | 12.87 | 10.85 | 4.83 | 12.08 |
| **36** | *L. purpuraventra* | 10.18 | 9.34–12.19 | 12.05 | 9.73 | 11.29–11.55 | 10.21 | 9.21 | 12.43 | 10.38 | 11.39 | 13.06 | 11.45 | 9.95 | 2.91 | 11.15 |
| **37** | *L. shangsiensis* | 7.18 | 6.75–8.03 | 8.56 | 6.40–7.25 | 8.77–9.45 | 7.09 | 7.97 | 8.98 | 8.68 | 11.20 | 8.56 | 9.22 | 8.96 | 12.07 | 9.37 |
| **38** | *L. mangshanensis* | 10.05 | 9.62–11.77 | 9.77 | 8.96–9.44 | 11.50–12.41 | 11.02 | 11.45 | 11.86 | 11.64 | 12.14 | 13.14 | 11.27 | 5.91 | 10.75 | 12.17 |
| **39** | *L. yunkaiensis* | 10.53 | 9.02–10.59 | 11.30 | 9.20–9.66 | 11.80–12.59 | 10.10 | 10.53 | 12.71 | 11.54 | 11.24 | 12.25 | 11.91 | 6.44 | 9.63 | 12.56 |
| **40** | *L. sola* | 18.50 | 19.09–23.00 | 21.69 | 19.37–19.85 | 20.25–20.27 | 18.62 | 19.21 | 20.36 | 20.83 | 21.48 | 22.47 | 18.40 | 20.81 | 21.44 | 20.50 |
| **41** | *L. melanoleuca* | 11.66 | 12.37–14.96 | 12.68 | 10.53–11.95 | 12.88–13.69 | 10.54 | 12.34 | 12.68 | 12.92 | 12.91 | 14.94 | 13.07 | 14.25 | 13.05 | 13.60 |
| **42** | *L. tuberosa* | 11.69 | 12.94–15.42 | 14.29 | 11.71–12.42 | 12.50–12.70 | 11.49 | 12.87 | 12.88 | 12.05 | 13.21 | 14.91 | 13.62 | 11.71 | 14.27 | 12.20 |
| **43** | *L. crocea* | 12.18 | 12.62–14.66 | 15.04 | 12.70–12.94 | 14.55–15.50 | 12.77 | 13.82 | 14.09 | 13.70 | 15.17 | 14.44 | 13.80 | 12.43 | 14.99 | 14.12 |
| **44** | *L. botsfordi* | 10.40 | 11.62–13.37 | 12.66 | 10.52–11.45 | 11.95–12.43 | 10.46 | 11.54 | 11.77 | 10.65 | 12.09 | 13.72 | 12.98 | 10.41 | 14.59 | 12.36 |
| **45** | *L. maoershanensis* | 10.09 | 9.41–11.18 | 10.37 | 9.00–9.23 | 10.37–11.29 | 10.11 | 10.08 | 11.72 | 11.00 | 11.69 | 12.50 | 11.94 | 5.00 | 10.91 | 11.51 |
| **46** | *L. shimentaina* | 9.72 | 8.82–9.87 | 10.20 | 8.40 | 11.00–12.17 | 10.00 | 10.65 | 11.86 | 11.54 | 11.57 | 12.63 | 11.00 | 5.28 | 9.28 | 11.60 |
| **47** | *L. tengchongensis* | 9.96 | 10.27–12.78 | 9.45 | 9.27–10.42 | 11.17–11.40 | 10.23 | 9.91 | 11.38 | 9.97 | 10.68 | 11.78 | 11.96 | 9.42 | 8.16 | 10.45 |
| **48** | *L. alpina* | 9.48 | 10.24–12.41 | 12.60 | 9.69–10.15 | 11.14–11.61 | 10.42 | 9.87 | 12.95 | 9.68 | 11.63 | 12.21 | 11.94 | 8.11 | 4.60 | 9.95 |
| **49** | *L. macrops* | 14.48 | 15.43–19.75 | 17.91 | 14.84–15.41 | 16.93–17.21 | 16.08 | 14.70 | 17.09 | 17.95 | 16.08 | 14.75 | 16.57 | 15.48 | 15.50 | 17.79 |
| **50** | *L. rowleyae* | 12.30 | 13.08–15.88 | 15.38 | 12.56–13.33 | 14.84–15.59 | 14.02 | 13.58 | 14.35 | 14.51 | 16.67 | 13.83 | 14.33 | 13.08 | 13.63 | 14.75 |
| **51** | *L. purpurus* | 9.97 | 9.36–12.23 | 11.47 | 9.95–10.39 | 11.59–12.09 | 9.79 | 9.50 | 12.05 | 9.53 | 9.81 | 12.22 | 11.01 | 7.77 | 3.79 | 9.57 |
| **52** | *L. namdongensis* | 10.84 | 10.80–13.42 | 9.99 | 9.35–10.45 | 10.43–11.07 | 8.81 | 9.93 | 10.16 | 10.44 | 9.94 | 12.11 | 12.20 | 8.72 | 9.54 | 11.15 |
| **53** | *L. kecil* | 16.49 | 18.29–21.15 | 20.61 | 18.29–18.32 | 19.70–19.92 | 18.49 | 19.50 | 19.48 | 19.52 | 19.23 | 18.78 | 18.17 | 18.49 | 19.43 | 19.77 |
| **54** | *L. kajangensis* | 14.04 | 14.14–17.44 | 18.00 | 13.37–14.00 | 15.53–15–74 | 15.35 | 14.80 | 15.48 | 16.39 | 17.20 | 16.59 | 14.37 | 14.85 | 17.24 | 15.85 |
| **55** | *L. itiokai* | 17.55 | 19.15–24.47 | 19.08 | 17.96–18.29 | 18.45–19.37 | 17.74 | 17.79 | 19.06 | 18.82 | 19.24 | 18.28 | 17.78 | 18.01 | 19.16 | 17.66 |
| **56** | *L. baluensis* | 15.65 | 16.63–21.02 | 20.18 | 16.90–17.22 | 17.10–17.98 | 18.06 | 17.87 | 17.80 | 18.97 | 16.73 | 19.14 | 17.00 | 18.03 | 17.55 | 18.98 |
| **57** | *L. parva* | 15.44 | 16.10–20.12 | 17.29 | 16.28–16.54 | 16.89–17.17 | 15.54 | 15.42 | 16.72 | 16.60 | 15.10 | 17.18 | 16.90 | 16.44 | 17.92 | 15.57 |
| **58** | *L. puhoatensis* | 11.48 | 10.21–12.54 | 11.21 | 10.65–11.07 | 12.15–12.35 | 8.86 | 11.80 | 11.08 | 12.02 | 10.42 | 11.85 | 12.01 | 10.45 | 9.75 | 12.59 |
| **59** | *L. petrops* | 11.40 | 11.15–13.88 | 12.63 | 9.98–10.83 | 13.08–13.32 | 10.46 | 11.09 | 12.22 | 11.56 | 10.58 | 13.21 | 13.88 | 10.91 | 10.23 | 12.33 |
| **60** | *L. khasiorum* | 13.43 | 13.43–14.82 | 14.08 | 13.28–13.52 | 15.25–15.57 | 12.11 | 14.66 | 15.90 | 14.42 | 14.75 | 15.75 | 14.56 | 11.90 | 12.77 | 15.85 |
| **61** | *L. isos* | 10.83 | 12.66–15.09 | 13.36 | 10.63–11.10 | 12.02–12.45 | 10.76 | 12.16 | 12.33 | 12.02 | 12.75 | 12.71 | 11.66 | 12.21 | 11.62 | 12.48 |
| **62** | *L. fuliginosa* | 10.91 | 12.00–15.11 | 14.23 | 11.99–13.09 | 13.42–13.87 | 11.83 | 12.95 | 13.54 | 13.88 | 12.48 | 14.54 | 12.32 | 14.28 | 14.12 | 14.80 |
| **63** | *L. bourreti* | 10.23 | 10.24–12.98 | 12.03 | 9.90 | 11.82–12.10 | 8.56 | 9.88 | 12.64 | 10.15 | 11.60 | 12.32 | 9.99 | 8.83 | 2.50 | 11.14 |
| **64** | *L. tadungensis* | 12.68 | 12.75–15.27 | 16.38 | 13.40–13.90 | 14.92–15.14 | 13.89 | 15.06 | 14.61 | 16.15 | 15.33 | 15.09 | 14.34 | 14.11 | 16.65 | 16.13 |
| **65** | *L. maculosa* | 13.08 | 14.33–17.76 | 17.11 | 13.50–14.00 | 15.02–15.50 | 14.22 | 14.13 | 14.47 | 15.02 | 14.75 | 14.76 | 13.37 | 14.05 | 15.76 | 15.73 |
| **66** | *L. kalonensis* | 14.71 | 15.66–19.30 | 18.12 | 15.39–16.10 | 18.37–18.39 | 15.76 | 16.86 | 16.66 | 16.98 | 16.18 | 17.27 | 15.98 | 16.05 | 17.71 | 17.45 |
| **67** | *L. pallida* | 14.91 | 15.49–19.10 | 16.50 | 14.97–15.68 | 18.10–18.38 | 15.79 | 15.88 | 15.63 | 17.02 | 16.78 | 15.98 | 16.15 | 16.26 | 17.27 | 16.91 |
| **68** | *L. ardens* | 12.25 | 14.29–17.76 | 16.11 | 12.90–13.14 | 15.09–15.32 | 14.29 | 14.18 | 13.55 | 14.32 | 15.56 | 14.38 | 13.14 | 12.27 | 14.58 | 14.78 |
| **69** | *L. pyrrhops* | 17.02 | 18.69–21.33 | 20.30 | 17.35–18.19 | 19.02–19.05 | 17.67 | 17.53 | 16.88 | 19.55 | 19.06 | 18.97 | 16.52 | 17.31 | 19.40 | 19.98 |
| **70** | *L. liui* | 9.94 | 9.70–9.85 | 10.62 | 7.94–8.55 | 10.30–11.79 | 10.58 | 10.49 | 12.60 | 12.29 | 12.01 | 13.64 | 10.97 | 8.39 | 9.97 | 12.34 |
| **71** | *L. laui* | 11.60 | 11.34–11.57 | 11.95 | 9.98–10.55 | 12.63–13.81 | 11.70 | 13.08 | 13.43 | 13.35 | 13.98 | 14.07 | 14.87 | 7.40 | 12.27 | 13.42 |
| **72** | *L. picta* | 15.52 | 16.06–19.08 | 17.58 | 15.04–15.71 | 16.98–17.06 | 16.00 | 16.42 | 16.98 | 17.69 | 17.20 | 19.03 | 18.17 | 16.11 | 17.61 | 19.01 |
| **73** | *L. zhangyapingi* | 8.31 | 9.82–12.05 | 11.26 | 9.85–10.27 | 9.89–10.75 | 10.10 | 9.79 | 10.76 | 11.29 | 7.02 | 10.87 | 12.01 | 10.71 | 9.69 | 11.03 |
| **74** | *L. firthi* | 12.61 | 13.14–15.52 | 14.00 | 11.81–12.02 | 12.66–13.17 | 11.05 | 13.44 | 12.93 | 14.53 | 12.56 | 15.91 | 13.64 | 12.85 | 12.82 | 14.77 |
| **75** | *L. eos* | 10.89 | 10.22–12.95 | 12.85 | 10.67 | 12.14–12.85 | 11.17 | 11.33 | 14.47 | 10.70 | 10.70 | 13.65 | 11.69 | 9.31 | 4.84 | 12.62 |
| **76** | *L. bidoupensis* | 14.66 | 15.65–19.07 | 17.85 | 15.23–15.58 | 17.82–18.06 | 15.75 | 16.36 | 16.59 | 16.52 | 14.72 | 16.04 | 15.26 | 14.70 | 16.79 | 16.70 |
| **77** | *L. melica* | 11.43 | 12.83–16.03 | 15.15 | 11.58–12.52 | 14.02–14.50 | 12.60 | 12.02 | 13.37 | 12.62 | 14.34 | 13.46 | 13.40 | 13.47 | 14.15 | 13.32 |
| **78** | *L. applebyi* | 13.31 | 14.71–18.43 | 16.56 | 14.53–14.73 | 16.75–16.76 | 15.74 | 15.41 | 15.91 | 15.23 | 16.27 | 15.58 | 14.99 | 15.09 | 15.80 | 16.19 |
| **79** | *L. marmorata* | 14.66 | 15.47–18.75 | 16.66 | 14.30–14.72 | 15.25–15.28 | 14.47 | 15.20 | 16.03 | 15.61 | 15.08 | 16.06 | 14.52 | 15.48 | 16.71 | 17.11 |
| **80** | *L. hamidi* | 14.35 | 16.28–19.52 | 16.37 | 14.40–14.88 | 15.60–15.62 | 14.69 | 16.16 | 16.23 | 15.09 | 16.27 | 15.95 | 15.44 | 14.95 | 16.62 | 16.58 |
| **81** | *L. gracilis* | 18.51 | 18.80–22.75 | 19.94 | 18.54–19.27 | 20.52–20.80 | 17.95 | 18.61 | 19.30 | 18.80 | 20.14 | 20.21 | 19.52 | 17.90 | 21.01 | 19.51 |
| **82** | *L. maura* | 15.44 | 16.64–20.33 | 16.89 | 15.38–16.09 | 16.68–16.88 | 14.76 | 15.96 | 16.38 | 16.59 | 16.16 | 16.26 | 16.30 | 15.53 | 16.43 | 16.47 |
| **83** | *L. arayai* | 16.08 | 17.16–20.38 | 16.39 | 16.37–16.40 | 16.16–17.14 | 14.97 | 15.96 | 17.51 | 16.89 | 15.86 | 16.59 | 14.72 | 15.07 | 16.75 | 17.64 |
| **84** | *L. fritinniens* | 15.04 | 15.62–18.68 | 17.23 | 14.86–15.57 | 15.37–15.66 | 15.75 | 15.96 | 16.77 | 16.16 | 16.90 | 18.80 | 16.95 | 16.36 | 17.71 | 16.33 |
| **85** | *L. dringi* | 15.20 | 15.20–18.40 | 16.83 | 14.73–15.89 | 15.70–15.74 | 14.23 | 15.47 | 16.59 | 16.31 | 16.40 | 18.40 | 17.66 | 15.58 | 16.30 | 17.09 |
| **86** | *L. sabahmontana* | 14.89 | 15.40–19.28 | 16.01 | 14.65–15.80 | 15.12–15.18 | 14.75 | 15.69 | 16.24 | 16.03 | 16.92 | 17.36 | 16.91 | 15.59 | 16.77 | 18.14 |
| **87** | *L. heteropus* | 18.14 | 18.17–20.16 | 21.54 | 18.92–18.96 | 20.92–20.94 | 20.14 | 20.40 | 20.38 | 20.21 | 22.38 | 22.16 | 20.42 | 21.00 | 22.23 | 21.02 |

|  | **16** | **17** | **18** | **19** | **20** | **21** | **22** | **23** | **24** | **25** | **26** | **27** | **28** | **29** | **30** | **31** | **32** | **33** |
| --- | --- | --- | --- | --- | --- | --- | --- | --- | --- | --- | --- | --- | --- | --- | --- | --- | --- | --- |
| **16** | **0.00** |  |  |  |  |  |  |  |  |  |  |  |  |  |  |  |  |  |
| **17** | 3.13–3.54 | **0.00–0.77** |  |  |  |  |  |  |  |  |  |  |  |  |  |  |  |  |
| **18** | 9.04 | 10.64–11.04 | **0.00** |  |  |  |  |  |  |  |  |  |  |  |  |  |  |  |
| **19** | 9.64 | 11.34–12.07 | 14.96 | **0.00** |  |  |  |  |  |  |  |  |  |  |  |  |  |  |
| **20** | 12.51 | 12.18–13.10 | 17.31 | 12.20 | **0.00** |  |  |  |  |  |  |  |  |  |  |  |  |  |
| **21** | 12.23 | 13.60–14.32 | 15.55 | 11.17 | 7.85 | **0.00** |  |  |  |  |  |  |  |  |  |  |  |  |
| **22** | 8.48 | 9.74–10.97 | 13.84 | 6.68 | 11.95 | 11.93 | **0.00** |  |  |  |  |  |  |  |  |  |  |  |
| **23** | 11.13 | 11.66–12.61 | 13.73 | 10.45 | 10.73 | 11.19 | 7.19 | **0.00** |  |  |  |  |  |  |  |  |  |  |
| **24** | 8.86 | 9.79–10.23 | 12.29 | 8.01 | 11.17 | 9.53 | 6.16 | 8.33 | **0.00** |  |  |  |  |  |  |  |  |  |
| **25** | 9.55–10.68 | 11.51–12.82 | 12.87–14.07 | 5.55–5.89 | 10.69–11.85 | 10.38–10.98 | 6.38–6.57 | 9.55–9.83 | 6.86–7.36 | **0.00–0.86** |  |  |  |  |  |  |  |  |
| **26** | 8.92 | 9.13–9.84 | 12.44 | 6.03 | 10.55 | 8.82 | 6.20 | 9.68 | 6.64 | 4.46–4.69 | **0.00** |  |  |  |  |  |  |  |
| **27** | 8.40 | 9.13–9.80 | 11.97 | 5.32 | 10.97 | 10.00 | 6.14 | 9.49 | 6.07 | 3.57–3.94 | 4.43 | **0.00** |  |  |  |  |  |  |
| **28** | 8.62 | 9.33–10.04 | 12.82 | 2.39 | 11.02 | 9.23 | 5.53 | 9.18 | 6.37 | 4.42–4.65 | 3.76 | 3.54 | **0.00** |  |  |  |  |  |
| **29** | 12.12 | 13.71–14.34 | 13.73 | 15.99 | 15.82 | 16.53 | 14.72 | 14.22 | 14.93 | 14.44–15.78 | 13.51 | 14.22 | 14.30 | **0.00** |  |  |  |  |
| **30** | 8.39 | 9.24–9.68 | 12.48 | 5.70 | 10.92 | 9.91 | 6.58 | 8.10 | 6.98 | 4.38–4.61 | 3.13 | 4.79 | 4.17 | 12.26 | **0.00** |  |  |  |
| **31** | 8.51 | 8.99–10.34 | 11.82–12.88 | 12.48–12.52 | 12.90–14.20 | 11.55–12.92 | 9.70–10.37 | 12.13 | 10.13–10.17 | 10.70–12.37 | 9.24 | 10.15–10.18 | 10.40 | 13.08–14.33 | 8.74–9.32 | **0.00** |  |  |
| **32** | 8.16 | 8.88–9.59 | 11.65 | 4.86 | 10.59 | 9.80 | 4.90 | 9.21 | 6.21 | 5.20–5.37 | 4.86 | 4.65 | 3.11 | 14.23 | 5.72 | 10.72 | **0.00** |  |
| **33** | 8.42 | 9.59–10.30 | 12.43 | 2.38 | 11.80 | 10.47 | 6.87 | 10.15 | 7.94 | 6.03–6.19 | 6.36 | 5.71 | 2.89 | 13.84 | 5.48 | 11.37 | 5.70 | **0.00** |
| **34** | 10.27 | 12.12–12.82 | 14.54 | 5.30 | 11.56 | 11.06 | 7.08 | 9.20 | 7.15 | 5.32–5.47 | 5.70 | 5.26 | 3.32 | 15.25 | 5.86 | 12.32–12.88 | 5.71 | 5.05 |
| **35** | 8.66 | 11.19–11.87 | 14.11 | 1.65 | 14.09 | 12.36 | 7.27 | 10.11 | 8.03 | 6.73–7.18 | 5.93 | 5.26 | 2.06 | 16.69 | 6.05 | 11.16–11.80 | 4.82 | 2.06 |
| **36** | 8.41 | 10.71–11.38 | 12.42 | 3.35 | 10.66 | 9.94 | 6.24 | 8.49 | 6.08 | 4.55–4.78 | 3.96 | 4.20 | 1.85 | 14.23 | 4.55 | 10.12–10.54 | 4.62 | 3.12 |
| **37** | 9.49 | 9.53–9.95 | 3.17 | 14.67 | 17.04 | 15.18 | 13.26 | 13.73 | 12.28 | 12.75–13.71 | 12.64 | 12.42 | 12.79 | 15.21 | 12.42 | 12.04–12.40 | 12.39 | 12.87 |
| **38** | 5.74 | 4.87–5.06 | 11.20 | 12.57 | 14.72 | 14.79 | 11.03 | 12.88 | 10.75 | 11.97–12.81 | 10.79 | 10.95 | 11.68 | 14.54 | 10.38 | 10.18–10.64 | 11.29 | 11.72 |
| **39** | 5.82 | 6.26–6.93 | 10.53 | 12.48 | 15.02 | 13.42 | 10.19 | 12.90 | 10.83 | 10.09–10.74 | 10.66 | 9.84 | 10.34 | 15.01 | 9.63 | 8.92–8.94 | 9.68 | 11.08 |
| **40** | 20.33 | 21.18–21.91 | 20.73 | 23.76 | 25.14 | 24.32 | 21.79 | 21.99 | 21.57 | 22.37–24.05 | 22.31 | 22.29 | 22.01 | 17.87 | 20.29 | 20.84 | 22.44 | 22.17 |
| **41** | 14.33 | 14.35–14.56 | 14.47 | 14.03 | 16.14 | 14.71 | 13.79 | 14.35 | 13.02 | 14.59–15.49 | 13.39 | 13.08 | 12.48 | 8.01 | 12.52 | 14.97 | 14.20 | 13.06 |
| **42** | 10.83 | 12.11–12.80 | 13.78 | 16.19 | 17.31 | 16.67 | 13.27 | 14.19 | 14.12 | 13.77–15.20 | 13.82 | 13.60 | 14.28 | 10.83 | 13.26 | 13.43 | 13.80 | 15.52 |
| **43** | 13.25 | 14.06–14.27 | 15.55 | 16.12 | 17.46 | 18.10 | 15.30 | 16.94 | 16.35 | 15.62–16.63 | 14.60 | 15.00 | 15.43 | 11.69 | 13.21 | 13.33 | 14.91 | 14.97 |
| **44** | 10.24 | 10.07–10.28 | 13.39 | 14.94 | 15.16 | 15.44 | 14.82 | 15.62 | 12.69 | 14.40–15.53 | 13.54 | 13.44 | 14.09 | 9.10 | 12.59 | 13.32 | 13.36 | 13.34 |
| **45** | 6.62 | 6.39–6.59 | 10.52 | 13.09 | 14.81 | 13.77 | 10.96 | 12.98 | 10.93 | 11.70–12.54 | 11.18 | 11.15 | 12.07 | 14.47 | 10.66 | 9.33 | 11.20 | 12.34 |
| **46** | 5.30 | 5.08–5.49 | 9.97 | 11.09 | 14.07 | 12.79 | 9.79 | 11.81 | 9.76 | 10.73–11.17 | 9.77 | 9.74 | 10.43 | 13.59 | 9.04 | 8.95 | 10.26 | 10.46 |
| **47** | 9.79 | 9.30–9.55 | 10.95 | 10.05 | 10.80 | 10.80 | 7.56 | 9.45 | 8.93 | 8.68–9.13 | 8.37 | 8.88 | 7.94 | 12.34 | 9.30 | 9.18 | 7.98 | 9.77 |
| **48** | 8.38 | 8.62–9.32 | 11.20 | 6.21 | 11.25 | 9.24 | 6.44 | 9.20 | 7.48 | 5.67–5.80 | 4.82 | 5.69 | 4.57 | 12.79 | 4.40 | 10.16 | 5.24 | 5.25 |
| **49** | 16.22 | 15.94–17.10 | 18.03 | 19.79 | 19.30 | 18.16 | 16.93 | 16.26 | 17.04 | 18.07–19.02 | 16.36 | 17.96 | 17.56 | 15.00 | 16.15 | 15.93 | 18.15 | 18.54 |
| **50** | 13.78 | 15.16–15.69 | 15.99 | 15.59 | 15.81 | 16.50 | 13.11 | 13.81 | 13.37 | 13.27–15.09 | 13.39 | 13.94 | 14.67 | 12.36 | 12.07 | 13.95 | 14.08 | 14.44 |
| **51** | 7.95 | 9.36–10.04 | 11.41 | 4.91 | 11.70 | 9.78 | 6.19 | 8.35 | 5.92 | 5.39–5.54 | 3.80 | 4.66 | 3.56 | 13.72 | 4.18 | 9.00–9.20 | 4.23 | 5.52 |
| **52** | 8.79 | 8.61–9.03 | 11.48 | 11.03 | 14.39 | 14.05 | 10.74 | 13.10 | 11.38 | 9.74–10.32 | 9.32 | 10.43 | 9.48 | 14.20 | 9.39 | 9.81–10.95 | 9.57 | 9.96 |
| **53** | 19.90 | 19.89–21.02 | 20.81 | 23.02 | 21.57 | 24.46 | 21.13 | 22.40 | 20.53 | 21.51–22.04 | 20.76 | 21.34 | 21.21 | 16.95 | 19.64 | 18.71–19.85 | 21.73 | 20.54 |
| **54** | 16.12 | 17.20–17.85 | 15.97 | 19.87 | 18.71 | 20.07 | 18.34 | 18.00 | 18.19 | 18.41–20.27 | 18.27 | 18.65 | 17.78 | 12.34 | 17.24 | 16.66–17.82 | 17.95 | 18.69 |
| **55** | 19.81 | 20.14–20.71 | 20.86 | 23.19 | 22.35 | 19.84 | 21.04 | 18.73 | 18.89 | 21.23–23.56 | 19.89 | 21.63 | 20.22 | 16.64 | 19.41 | 19.02 | 21.76 | 21.42 |
| **56** | 18.99 | 18.13–18.68 | 19.23 | 21.80 | 20.52 | 20.28 | 19.68 | 17.04 | 18.76 | 21.18–22.76 | 19.18 | 19.68 | 19.50 | 14.26 | 17.75 | 17.81 | 19.50 | 20.22 |
| **57** | 17.90 | 17.56–18.92 | 18.47 | 21.31 | 20.32 | 19.40 | 20.51 | 17.94 | 16.78 | 19.17–20.77 | 18.61 | 19.91 | 19.19 | 15.90 | 18.47 | 17.49–17.89 | 20.25 | 20.00 |
| **58** | 9.49 | 9.79–10.12 | 12.31 | 11.94 | 12.94 | 15.54 | 13.11 | 12.32 | 11.74 | 11.51–12.74 | 9.97 | 11.07 | 10.14 | 14.76 | 9.60 | 9.09–10.70 | 11.42 | 10.63 |
| **59** | 9.05 | 9.62–9.97 | 12.70 | 13.20 | 12.88 | 14.22 | 12.25 | 13.01 | 12.05 | 12.03–13.33 | 9.99 | 11.56 | 11.12 | 14.26 | 10.79 | 7.43–8.62 | 11.23 | 12.32 |
| **60** | 11.73 | 11.65–12.17 | 14.25 | 13.91 | 15.11 | 14.57 | 13.60 | 15.28 | 12.61 | 11.67–11.70 | 11.94 | 13.66 | 14.08 | 15.10 | 12.27 | 9.17–10.17 | 12.54 | 14.04 |
| **61** | 12.01 | 12.90–13.61 | 12.67 | 15.18 | 16.09 | 16.33 | 13.94 | 15.04 | 13.16 | 13.13–13.28 | 13.58 | 13.43 | 13.10 | 12.61 | 12.97 | 13.30–14.57 | 12.76 | 13.84 |
| **62** | 13.11 | 13.97–14.40 | 14.79 | 17.05 | 17.44 | 17.69 | 15.39 | 15.69 | 14.42 | 14.86–15.98 | 13.73 | 14.17 | 14.84 | 8.51 | 13.66 | 13.03–14.31 | 15.84 | 15.68 |
| **63** | 9.54 | 10.59–11.24 | 11.66 | 4.90 | 11.13 | 10.14 | 6.10 | 8.12 | 6.53 | 3.05–3.39 | 3.79 | 3.61 | 3.13 | 13.25 | 3.74 | 9.90–10.56 | 4.02 | 5.08 |
| **64** | 13.91 | 14.47–14.69 | 16.34 | 17.07 | 18.04 | 17.85 | 17.07 | 16.62 | 15.68 | 15.64–16.85 | 16.20 | 16.45 | 16.60 | 13.04 | 14.96 | 15.18–15.67 | 16.60 | 17.17 |
| **65** | 13.78 | 15.12–15.29 | 15.22 | 18.07 | 17.65 | 17.55 | 17.58 | 15.69 | 16.16 | 16.11–17.71 | 14.99 | 16.16 | 16.74 | 12.01 | 14.66 | 14.84–15.68 | 16.18 | 16.50 |
| **66** | 15.99 | 17.01–17.58 | 17.80 | 17.94 | 19.37 | 18.51 | 19.06 | 17.42 | 15.46 | 18.46–20.34 | 16.44 | 17.50 | 17.41 | 14.27 | 15.82 | 15.63–16.14 | 16.84 | 17.92 |
| **67** | 15.00 | 17.49–18.00 | 18.59 | 17.79 | 17.85 | 17.03 | 18.37 | 16.37 | 15.51 | 17.50–19.57 | 14.91 | 16.77 | 16.97 | 14.81 | 16.12 | 15.36–15.88 | 16.97 | 17.77 |
| **68** | 14.09 | 14.86–15.50 | 15.31 | 17.58 | 15.15 | 15.12 | 15.41 | 14.03 | 14.22 | 15.32–17.08 | 14.28 | 14.70 | 15.54 | 10.78 | 13.24 | 14.33–14.87 | 15.48 | 15.59 |
| **69** | 17.20 | 18.45–18.72 | 20.94 | 19.76 | 19.40 | 18.82 | 19.80 | 19.25 | 17.72 | 18.99–20.09 | 18.62 | 18.54 | 20.40 | 16.49 | 18.64 | 17.21–17.44 | 20.11 | 20.98 |
| **70** | 6.73 | 5.90–6.45 | 12.73 | 12.44 | 15.75 | 13.16 | 11.64 | 14.52 | 10.66 | 10.46–11.35 | 11.28 | 11.16 | 12.05 | 16.18 | 9.74 | 10.22 | 11.51 | 12.39 |
| **71** | 7.77 | 6.09–6.63 | 11.98 | 13.57 | 14.07 | 13.15 | 12.45 | 16.27 | 13.56 | 11.97–12.26 | 12.60 | 10.77 | 12.88 | 16.91 | 11.72 | 9.51 | 12.32 | 12.93 |
| **72** | 17.91 | 18.03–18.37 | 17.66 | 20.55 | 19.18 | 21.02 | 18.20 | 18.94 | 17.64 | 17.34–18.23 | 17.66 | 19.46 | 19.10 | 15.55 | 18.30 | 17.99–19.16 | 18.85 | 19.06 |
| **73** | 9.93 | 11.20–11.85 | 11.34 | 13.37 | 16.24 | 14.26 | 11.26 | 13.15 | 12.45 | 11.50–11.72 | 10.86 | 11.50 | 11.55 | 13.94 | 11.25 | 12.04–13.10 | 11.77 | 11.15 |
| **74** | 12.54 | 13.14–13.37 | 13.82 | 15.92 | 17.04 | 18.17 | 14.60 | 16.31 | 14.84 | 13.29–13.76 | 13.54 | 14.59 | 14.32 | 15.49 | 14.15 | 15.03–16.23 | 13.78 | 14.06 |
| **75** | 8.40 | 9.17–9.86 | 13.10 | 7.07 | 12.31 | 10.29 | 6.35 | 10.46 | 6.95 | 4.57–4.80 | 4.23 | 5.64 | 5.27 | 14.89 | 5.03 | 9.47–9.76 | 4.88 | 7.93 |
| **76** | 14.93 | 16.38–16.77 | 17.38 | 18.53 | 19.42 | 18.80 | 18.15 | 16.68 | 16.77 | 17.70–18.94 | 16.77 | 18.06 | 17.99 | 13.10 | 15.05 | 15.32–15.84 | 16.67 | 17.99 |
| **77** | 13.87 | 15.24–15.58 | 14.65 | 15.81 | 16.30 | 17.90 | 13.61 | 14.51 | 14.01 | 14.20–15.83 | 14.01 | 14.49 | 14.33 | 11.63 | 13.00 | 14.69–15.25 | 14.27 | 13.84 |
| **78** | 14.79 | 15.88–16.47 | 16.28 | 17.83 | 17.11 | 16.22 | 16.35 | 15.14 | 15.14 | 16.31–17.64 | 16.27 | 16.44 | 17.09 | 12.75 | 15.11 | 15.78–16.32 | 16.27 | 15.52 |
| **79** | 16.33 | 17.02–17.39 | 17.01 | 18.61 | 18.66 | 19.41 | 17.75 | 16.76 | 15.62 | 16.61–17.69 | 15.87 | 18.14 | 16.67 | 14.45 | 16.22 | 15.80–16.99 | 16.69 | 16.89 |
| **80** | 16.34 | 16.77–17.30 | 16.90 | 19.07 | 17.60 | 19.16 | 17.42 | 17.73 | 17.07 | 16.78–17.88 | 15.78 | 17.82 | 17.07 | 13.73 | 16.64 | 14.71–15.94 | 16.34 | 17.40 |
| **81** | 18.96 | 19.53–20.01 | 20.13 | 23.20 | 21.56 | 23.46 | 21.48 | 19.77 | 22.34 | 21.04–22.36 | 20.99 | 21.06 | 21.57 | 16.69 | 20.51 | 20.17–21.51 | 21.84 | 21.01 |
| **82** | 16.60 | 17.74–18.56 | 16.44 | 18.31 | 19.50 | 21.59 | 18.04 | 17.85 | 17.59 | 17.63–19.14 | 17.97 | 18.68 | 17.11 | 14.10 | 17.19 | 17.22–18.37 | 17.94 | 15.83 |
| **83** | 16.60 | 16.80–17.67 | 18.01 | 18.87 | 18.40 | 18.74 | 17.96 | 17.29 | 16.60 | 17.34–17.70 | 16.41 | 18.43 | 16.92 | 14.90 | 17.26 | 15.02–16.24 | 16.71 | 17.14 |
| **84** | 16.85 | 17.00–17.30 | 17.14 | 20.59 | 18.67 | 19.15 | 17.80 | 18.13 | 16.88 | 17.59–17.92 | 17.20 | 18.67 | 18.02 | 14.46 | 18.34 | 17.92–19.06 | 17.79 | 18.55 |
| **85** | 17.02 | 15.48–16.34 | 17.10 | 19.58 | 17.63 | 20.21 | 17.01 | 17.83 | 16.36 | 17.26–17.88 | 17.05 | 17.33 | 17.40 | 14.96 | 16.94 | 17.64–18.53 | 17.47 | 18.24 |
| **86** | 16.52 | 16.37–16.92 | 16.42 | 20.84 | 20.42 | 21.46 | 18.13 | 17.72 | 17.06 | 17.46–18.36 | 17.29 | 18.28 | 18.14 | 14.92 | 17.64 | 17.41–18.56 | 18.24 | 18.70 |
| **87** | 21.21 | 22.15–23.12 | 21.10 | 24.06 | 22.99 | 23.55 | 23.01 | 21.90 | 23.47 | 22.19–23.40 | 23.10 | 24.19 | 23.45 | 18.09 | 21.62 | 21.37–22.72 | 23.34 | 22.78 |

|  | **34** | **35** | **36** | **37** | **38** | **39** | **40** | **41** | **42** | **43** | **44** | **45** | **46** | **47** | **48** | **49** | **50** | **51** |
| --- | --- | --- | --- | --- | --- | --- | --- | --- | --- | --- | --- | --- | --- | --- | --- | --- | --- | --- |
| **34** | **0.00** |  |  |  |  |  |  |  |  |  |  |  |  |  |  |  |  |  |
| **35** | 5.97 | **0.00** |  |  |  |  |  |  |  |  |  |  |  |  |  |  |  |  |
| **36** | 3.91 | 4.15 | **0.00** |  |  |  |  |  |  |  |  |  |  |  |  |  |  |  |
| **37** | 14.50 | 13.89 | 12.64 | **0.00** |  |  |  |  |  |  |  |  |  |  |  |  |  |  |
| **38** | 13.00 | 11.90 | 11.64 | 10.92 | **0.00** |  |  |  |  |  |  |  |  |  |  |  |  |  |
| **39** | 11.30 | 11.55 | 10.11 | 11.21 | 7.15 | **0.00** |  |  |  |  |  |  |  |  |  |  |  |  |
| **40** | 22.15 | 23.13 | 21.71 | 21.26 | 22.57 | 21.28 | **0.00** |  |  |  |  |  |  |  |  |  |  |  |
| **41** | 13.39 | 13.48 | 11.95 | 14.47 | 15.17 | 16.89 | 18.98 | **0.00** |  |  |  |  |  |  |  |  |  |  |
| **42** | 14.68 | 15.52 | 15.00 | 13.78 | 13.82 | 14.18 | 20.21 | 12.62 | **0.00** |  |  |  |  |  |  |  |  |  |
| **43** | 14.68 | 15.45 | 14.69 | 15.53 | 15.58 | 15.19 | 19.73 | 13.79 | 11.96 | **0.00** |  |  |  |  |  |  |  |  |
| **44** | 15.03 | 14.65 | 13.33 | 13.84 | 12.78 | 12.80 | 18.84 | 10.67 | 10.30 | 9.79 | **0.00** |  |  |  |  |  |  |  |
| **45** | 13.52 | 12.60 | 11.36 | 10.49 | 5.96 | 7.12 | 21.10 | 15.53 | 13.64 | 14.66 | 11.86 | **0.00** |  |  |  |  |  |  |
| **46** | 11.84 | 10.48 | 10.21 | 9.94 | 3.56 | 7.13 | 21.68 | 14.82 | 13.29 | 14.69 | 12.41 | 6.39 | **0.00** |  |  |  |  |  |
| **47** | 9.74 | 9.07 | 7.68 | 11.12 | 10.26 | 10.84 | 21.87 | 12.72 | 12.99 | 15.37 | 12.18 | 10.86 | 9.48 | **0.00** |  |  |  |  |
| **48** | 5.22 | 5.25 | 4.58 | 12.10 | 10.07 | 10.36 | 21.39 | 12.96 | 13.95 | 14.13 | 12.82 | 10.92 | 9.06 | 7.72 | **0.00** |  |  |  |
| **49** | 17.15 | 18.79 | 16.31 | 17.15 | 15.95 | 17.58 | 21.46 | 16.35 | 12.75 | 13.85 | 15.91 | 15.74 | 15.01 | 16.33 | 16.85 | **0.00** |  |  |
| **50** | 13.76 | 14.93 | 13.04 | 16.60 | 15.45 | 15.27 | 20.70 | 13.11 | 11.43 | 11.92 | 12.52 | 15.25 | 14.83 | 14.40 | 13.25 | 10.09 | **0.00** |  |
| **51** | 5.03 | 5.28 | 4.22 | 10.70 | 8.98 | 9.17 | 22.31 | 11.84 | 11.84 | 13.48 | 12.22 | 9.10 | 7.72 | 6.91 | 1.66 | 16.03 | 13.31 | **0.00** |
| **52** | 11.75 | 10.85 | 9.69 | 11.74 | 9.97 | 9.04 | 21.97 | 13.60 | 13.39 | 14.22 | 11.20 | 9.37 | 9.89 | 8.14 | 9.74 | 17.73 | 14.80 | 9.82 |
| **53** | 19.32 | 23.57 | 19.39 | 21.09 | 21.18 | 19.46 | 10.83 | 17.03 | 19.99 | 19.17 | 18.00 | 19.84 | 20.38 | 20.26 | 18.96 | 23.09 | 20.07 | 20.67 |
| **54** | 18.20 | 19.86 | 16.56 | 17.52 | 18.75 | 16.81 | 12.65 | 14.53 | 14.98 | 14.74 | 13.88 | 16.87 | 17.96 | 17.66 | 17.20 | 17.68 | 16.74 | 19.14 |
| **55** | 19.54 | 21.47 | 19.86 | 21.12 | 21.36 | 22.67 | 20.48 | 18.28 | 17.23 | 18.37 | 18.71 | 20.97 | 20.20 | 18.12 | 19.85 | 18.98 | 17.98 | 19.45 |
| **56** | 18.29 | 20.41 | 18.03 | 18.64 | 19.64 | 19.85 | 18.17 | 17.55 | 16.53 | 16.71 | 16.29 | 20.36 | 19.72 | 16.72 | 18.77 | 16.72 | 15.80 | 18.11 |
| **57** | 18.02 | 21.29 | 18.37 | 17.88 | 18.16 | 19.32 | 17.20 | 16.65 | 16.16 | 17.25 | 15.00 | 18.41 | 19.04 | 15.98 | 18.55 | 18.66 | 17.58 | 18.53 |
| **58** | 13.15 | 12.88 | 10.60 | 11.99 | 10.87 | 11.15 | 23.35 | 12.85 | 14.36 | 14.69 | 11.65 | 10.76 | 9.67 | 8.56 | 10.64 | 16.85 | 14.27 | 11.11 |
| **59** | 12.77 | 13.83 | 11.24 | 12.61 | 10.89 | 10.23 | 22.44 | 13.25 | 13.85 | 13.62 | 13.11 | 10.35 | 10.39 | 9.04 | 11.58 | 17.02 | 14.45 | 10.89 |
| **60** | 14.41 | 15.90 | 13.51 | 14.49 | 12.50 | 11.08 | 24.76 | 16.18 | 15.98 | 14.03 | 12.22 | 12.48 | 11.15 | 11.48 | 12.79 | 20.11 | 16.80 | 13.12 |
| **61** | 14.10 | 15.74 | 13.58 | 13.61 | 13.32 | 13.95 | 19.93 | 14.42 | 11.74 | 14.11 | 12.93 | 13.18 | 13.70 | 10.91 | 12.82 | 15.93 | 16.20 | 13.43 |
| **62** | 15.89 | 17.18 | 15.39 | 15.05 | 15.00 | 15.21 | 17.01 | 6.20 | 11.13 | 12.89 | 11.25 | 14.27 | 14.32 | 14.48 | 14.53 | 14.35 | 12.03 | 14.60 |
| **63** | 4.83 | 5.80 | 3.34 | 11.51 | 11.41 | 9.82 | 21.93 | 12.82 | 13.33 | 15.09 | 13.38 | 11.36 | 9.98 | 7.78 | 4.20 | 17.29 | 12.84 | 4.23 |
| **64** | 16.44 | 17.45 | 16.95 | 15.28 | 13.33 | 14.80 | 20.07 | 13.12 | 11.08 | 11.46 | 11.71 | 15.04 | 13.38 | 15.85 | 16.31 | 8.45 | 9.02 | 14.92 |
| **65** | 17.09 | 18.19 | 16.36 | 16.60 | 13.73 | 15.43 | 19.14 | 14.49 | 10.57 | 12.68 | 11.82 | 15.22 | 13.22 | 15.41 | 15.15 | 8.19 | 9.23 | 16.06 |
| **66** | 17.17 | 18.54 | 16.98 | 16.84 | 15.40 | 18.09 | 21.66 | 15.46 | 12.88 | 11.32 | 13.07 | 16.43 | 14.95 | 16.68 | 16.80 | 8.76 | 11.31 | 15.68 |
| **67** | 16.72 | 19.10 | 16.56 | 17.40 | 15.64 | 16.77 | 20.09 | 14.91 | 11.87 | 11.36 | 13.15 | 16.45 | 14.98 | 16.24 | 16.60 | 7.68 | 9.61 | 15.74 |
| **68** | 14.77 | 16.99 | 14.04 | 15.96 | 15.50 | 16.00 | 18.63 | 12.80 | 10.50 | 10.88 | 10.74 | 14.24 | 14.82 | 14.69 | 14.47 | 8.64 | 6.73 | 15.29 |
| **69** | 20.32 | 22.36 | 19.11 | 19.43 | 17.45 | 18.43 | 17.81 | 17.22 | 12.98 | 15.19 | 14.68 | 17.62 | 17.38 | 18.64 | 19.95 | 10.21 | 11.59 | 18.54 |
| **70** | 13.29 | 13.31 | 11.79 | 11.47 | 3.69 | 8.22 | 24.25 | 17.20 | 14.38 | 16.36 | 13.64 | 7.57 | 4.51 | 11.81 | 11.18 | 16.87 | 16.50 | 9.49 |
| **71** | 14.12 | 13.26 | 13.79 | 11.63 | 7.48 | 8.98 | 26.25 | 18.64 | 14.63 | 16.68 | 13.46 | 8.25 | 5.54 | 10.77 | 11.10 | 20.08 | 16.99 | 10.34 |
| **72** | 17.77 | 21.41 | 18.62 | 18.13 | 19.05 | 20.30 | 20.57 | 16.23 | 14.58 | 16.54 | 15.40 | 18.51 | 19.08 | 16.82 | 18.96 | 16.44 | 16.05 | 19.84 |
| **73** | 11.74 | 12.66 | 11.67 | 11.85 | 11.82 | 11.91 | 22.15 | 13.88 | 13.42 | 14.39 | 12.55 | 12.43 | 11.57 | 10.39 | 9.90 | 17.22 | 16.62 | 10.18 |
| **74** | 15.69 | 15.71 | 14.76 | 15.04 | 14.33 | 14.68 | 22.05 | 17.33 | 15.74 | 15.16 | 14.78 | 14.99 | 14.20 | 12.33 | 14.04 | 18.75 | 16.96 | 14.36 |
| **75** | 6.68 | 7.35 | 5.60 | 13.04 | 10.99 | 9.13 | 22.27 | 14.52 | 12.56 | 14.23 | 12.89 | 10.43 | 9.75 | 8.90 | 5.93 | 16.57 | 14.09 | 3.51 |
| **76** | 17.46 | 19.31 | 17.52 | 17.17 | 15.09 | 15.65 | 19.63 | 14.67 | 10.84 | 10.32 | 12.31 | 15.40 | 14.95 | 16.71 | 15.88 | 7.64 | 10.79 | 15.71 |
| **77** | 13.91 | 15.58 | 12.68 | 15.03 | 16.55 | 15.84 | 19.77 | 12.51 | 11.01 | 10.00 | 10.75 | 15.56 | 16.11 | 14.71 | 13.99 | 10.31 | 6.76 | 15.12 |
| **78** | 16.45 | 17.76 | 15.71 | 17.20 | 17.27 | 17.29 | 18.28 | 13.94 | 12.06 | 11.81 | 12.03 | 17.09 | 16.32 | 17.24 | 15.98 | 11.58 | 9.81 | 16.32 |
| **79** | 16.56 | 19.24 | 15.92 | 16.41 | 17.18 | 17.77 | 19.64 | 14.93 | 12.47 | 14.28 | 12.93 | 16.87 | 16.89 | 15.76 | 16.81 | 14.78 | 13.63 | 17.26 |
| **80** | 16.49 | 20.12 | 16.76 | 15.11 | 17.41 | 18.22 | 20.40 | 14.34 | 12.97 | 14.80 | 14.18 | 16.03 | 17.40 | 15.17 | 16.21 | 15.50 | 14.58 | 17.19 |
| **81** | 20.02 | 23.52 | 21.21 | 19.95 | 20.83 | 20.70 | 21.58 | 17.38 | 15.90 | 17.15 | 17.47 | 19.80 | 20.30 | 19.51 | 20.35 | 19.57 | 18.58 | 23.04 |
| **82** | 16.43 | 18.70 | 17.17 | 17.05 | 18.45 | 18.51 | 19.05 | 14.76 | 13.53 | 15.31 | 15.80 | 16.59 | 17.89 | 16.57 | 16.60 | 16.49 | 16.07 | 18.27 |
| **83** | 16.56 | 19.49 | 15.89 | 16.94 | 17.17 | 18.34 | 20.48 | 13.89 | 13.23 | 14.80 | 14.68 | 15.86 | 17.18 | 15.01 | 16.82 | 14.18 | 13.87 | 17.30 |
| **84** | 16.61 | 20.83 | 18.65 | 16.56 | 18.49 | 19.09 | 19.96 | 14.90 | 12.69 | 16.00 | 14.36 | 18.46 | 18.97 | 16.86 | 17.38 | 15.84 | 16.39 | 18.06 |
| **85** | 15.85 | 20.28 | 17.30 | 17.29 | 17.61 | 19.21 | 20.23 | 15.26 | 13.73 | 14.72 | 14.04 | 17.29 | 17.86 | 16.24 | 17.26 | 15.67 | 16.48 | 18.19 |
| **86** | 17.19 | 20.72 | 18.22 | 16.79 | 18.11 | 19.23 | 20.15 | 15.00 | 13.19 | 16.61 | 14.97 | 17.03 | 18.10 | 16.49 | 18.26 | 14.47 | 15.05 | 19.13 |
| **87** | 23.50 | 25.94 | 22.14 | 20.42 | 23.10 | 22.52 | 6.70 | 20.13 | 21.37 | 21.08 | 20.53 | 22.83 | 21.50 | 22.14 | 22.51 | 22.81 | 20.62 | 23.99 |

|  | **52** | **53** | **54** | **55** | **56** | **57** | **58** | **59** | **60** | **61** | **62** | **63** | **64** | **65** | **66** | **67** | **68** | **69** |
| --- | --- | --- | --- | --- | --- | --- | --- | --- | --- | --- | --- | --- | --- | --- | --- | --- | --- | --- |
| **52** | **0.00** |  |  |  |  |  |  |  |  |  |  |  |  |  |  |  |  |  |
| **53** | 18.96 | **0.00** |  |  |  |  |  |  |  |  |  |  |  |  |  |  |  |  |
| **54** | 17.25 | 14.25 | **0.00** |  |  |  |  |  |  |  |  |  |  |  |  |  |  |  |
| **55** | 19.82 | 22.95 | 17.12 | **0.00** |  |  |  |  |  |  |  |  |  |  |  |  |  |  |
| **56** | 19.86 | 20.10 | 16.68 | 9.35 | **0.00** |  |  |  |  |  |  |  |  |  |  |  |  |  |
| **57** | 17.56 | 18.89 | 14.65 | 5.60 | 6.58 | **0.00** |  |  |  |  |  |  |  |  |  |  |  |  |
| **58** | 6.05 | 20.25 | 17.69 | 19.99 | 19.08 | 19.31 | **0.00** |  |  |  |  |  |  |  |  |  |  |  |
| **59** | 7.12 | 19.73 | 17.88 | 18.32 | 18.01 | 17.49 | 6.61 | **0.00** |  |  |  |  |  |  |  |  |  |  |
| **60** | 11.63 | 21.94 | 19.73 | 22.67 | 22.19 | 20.20 | 12.93 | 11.76 | **0.00** |  |  |  |  |  |  |  |  |  |
| **61** | 12.83 | 17.85 | 14.78 | 19.21 | 17.68 | 16.47 | 14.34 | 14.84 | 16.19 | **0.00** |  |  |  |  |  |  |  |  |
| **62** | 13.97 | 15.41 | 13.35 | 20.19 | 17.27 | 17.50 | 14.68 | 13.94 | 16.46 | 13.67 | **0.00** |  |  |  |  |  |  |  |
| **63** | 9.95 | 20.46 | 16.97 | 20.35 | 18.65 | 18.60 | 11.22 | 11.88 | 11.92 | 13.17 | 14.35 | **0.00** |  |  |  |  |  |  |
| **64** | 15.36 | 19.78 | 14.71 | 19.25 | 17.36 | 17.03 | 16.11 | 15.38 | 17.26 | 13.09 | 12.50 | 15.33 | **0.00** |  |  |  |  |  |
| **65** | 14.51 | 19.58 | 15.28 | 17.81 | 16.92 | 16.56 | 15.05 | 14.33 | 17.93 | 15.01 | 13.83 | 15.55 | 7.75 | **0.00** |  |  |  |  |
| **66** | 18.15 | 21.41 | 17.02 | 20.14 | 18.20 | 17.29 | 18.77 | 18.19 | 18.52 | 15.75 | 14.30 | 17.81 | 7.19 | 8.83 | **0.00** |  |  |  |
| **67** | 17.83 | 20.35 | 16.11 | 18.39 | 18.23 | 16.34 | 18.94 | 18.12 | 18.58 | 16.27 | 14.49 | 17.13 | 8.02 | 8.87 | 5.53 | **0.00** |  |  |
| **68** | 15.34 | 17.21 | 12.99 | 16.77 | 15.05 | 15.04 | 15.84 | 15.10 | 17.77 | 13.71 | 11.87 | 14.83 | 8.82 | 6.12 | 9.69 | 8.39 | **0.00** |  |
| **69** | 19.15 | 19.63 | 15.40 | 22.72 | 20.29 | 19.43 | 20.07 | 18.71 | 17.63 | 15.80 | 15.66 | 18.75 | 10.06 | 7.88 | 10.75 | 8.87 | 8.84 | **0.00** |
| **70** | 11.14 | 23.42 | 20.50 | 23.96 | 21.81 | 20.50 | 11.99 | 11.75 | 11.33 | 13.43 | 16.85 | 11.39 | 14.53 | 15.34 | 17.70 | 17.70 | 17.40 | 18.23 |
| **71** | 11.31 | 24.54 | 22.28 | 25.16 | 23.56 | 23.07 | 11.59 | 13.45 | 12.84 | 13.32 | 18.34 | 11.74 | 16.74 | 16.93 | 19.92 | 18.82 | 16.93 | 19.45 |
| **72** | 17.84 | 21.45 | 16.81 | 17.88 | 16.62 | 15.09 | 19.23 | 17.68 | 17.63 | 18.37 | 14.99 | 18.33 | 16.81 | 16.08 | 16.29 | 15.82 | 14.98 | 17.95 |
| **73** | 10.89 | 19.44 | 17.21 | 19.04 | 18.21 | 16.66 | 13.44 | 13.18 | 15.71 | 11.30 | 13.59 | 10.96 | 15.26 | 15.90 | 17.00 | 18.02 | 15.48 | 19.42 |
| **74** | 13.19 | 22.11 | 16.57 | 20.60 | 20.33 | 17.98 | 13.97 | 14.43 | 13.65 | 12.21 | 16.61 | 13.55 | 13.99 | 15.70 | 17.87 | 17.44 | 15.83 | 18.21 |
| **75** | 9.36 | 20.90 | 19.26 | 20.53 | 19.80 | 19.89 | 11.65 | 10.97 | 11.71 | 14.33 | 14.33 | 4.13 | 16.02 | 15.74 | 16.40 | 16.23 | 14.79 | 18.54 |
| **76** | 16.54 | 18.78 | 14.53 | 18.62 | 17.15 | 16.51 | 17.69 | 17.86 | 18.11 | 14.79 | 13.59 | 17.56 | 6.74 | 7.78 | 5.51 | 4.90 | 8.74 | 9.30 |
| **77** | 13.69 | 17.13 | 14.66 | 17.36 | 15.91 | 15.78 | 14.90 | 14.84 | 17.38 | 14.44 | 11.64 | 13.99 | 10.13 | 7.98 | 11.04 | 10.14 | 4.65 | 12.28 |
| **78** | 16.51 | 17.62 | 13.82 | 16.83 | 16.57 | 15.23 | 18.15 | 17.06 | 17.16 | 14.86 | 12.23 | 16.19 | 10.23 | 8.92 | 11.81 | 10.70 | 6.96 | 13.05 |
| **79** | 16.22 | 20.94 | 13.43 | 15.56 | 15.25 | 13.77 | 18.34 | 18.06 | 15.98 | 15.65 | 14.81 | 15.73 | 15.44 | 15.48 | 15.54 | 13.88 | 13.66 | 17.79 |
| **80** | 16.14 | 20.84 | 15.12 | 17.56 | 15.47 | 15.42 | 18.73 | 17.51 | 17.46 | 15.59 | 14.24 | 16.34 | 17.48 | 18.31 | 16.74 | 14.83 | 14.31 | 18.62 |
| **81** | 19.62 | 20.68 | 15.39 | 21.67 | 18.64 | 18.16 | 21.21 | 19.89 | 20.37 | 18.87 | 16.01 | 20.95 | 19.78 | 17.58 | 19.10 | 17.56 | 15.51 | 19.07 |
| **82** | 15.10 | 18.02 | 12.86 | 16.09 | 15.90 | 14.51 | 17.55 | 17.34 | 17.33 | 15.62 | 14.66 | 17.70 | 17.54 | 16.16 | 18.15 | 16.22 | 14.18 | 18.61 |
| **83** | 16.22 | 20.41 | 15.24 | 16.77 | 14.75 | 14.98 | 17.86 | 17.81 | 17.78 | 14.65 | 15.02 | 16.02 | 16.85 | 16.91 | 16.01 | 14.14 | 13.91 | 18.35 |
| **84** | 18.25 | 21.54 | 16.06 | 17.55 | 15.70 | 14.67 | 19.54 | 18.58 | 17.98 | 17.72 | 13.83 | 17.36 | 14.09 | 16.56 | 15.81 | 14.70 | 15.05 | 17.42 |
| **85** | 16.76 | 20.85 | 17.53 | 17.46 | 16.16 | 14.81 | 18.10 | 16.91 | 17.92 | 17.52 | 15.38 | 17.06 | 15.65 | 14.74 | 15.49 | 15.00 | 14.15 | 18.01 |
| **86** | 16.74 | 20.90 | 15.28 | 16.76 | 16.09 | 14.96 | 17.58 | 17.10 | 17.91 | 17.07 | 12.58 | 17.49 | 14.73 | 14.76 | 14.57 | 13.87 | 13.75 | 17.57 |
| **87** | 22.53 | 12.60 | 15.33 | 22.44 | 19.33 | 19.87 | 24.34 | 24.21 | 23.25 | 20.95 | 18.90 | 22.83 | 21.99 | 21.85 | 22.75 | 21.33 | 19.69 | 19.57 |

|  | **70** | **71** | **72** | **73** | **74** | **75** | **76** | **77** | **78** | **79** | **80** | **81** | **82** | **83** | **84** | **85** | **86** | **87** |
| --- | --- | --- | --- | --- | --- | --- | --- | --- | --- | --- | --- | --- | --- | --- | --- | --- | --- | --- |
| **70** | **0.00** |  |  |  |  |  |  |  |  |  |  |  |  |  |  |  |  |  |
| **71** | 7.45 | **0.00** |  |  |  |  |  |  |  |  |  |  |  |  |  |  |  |  |
| **72** | 20.97 | 22.34 | **0.00** |  |  |  |  |  |  |  |  |  |  |  |  |  |  |  |
| **73** | 13.14 | 12.98 | 18.38 | **0.00** |  |  |  |  |  |  |  |  |  |  |  |  |  |  |
| **74** | 14.77 | 15.59 | 17.13 | 13.17 | **0.00** |  |  |  |  |  |  |  |  |  |  |  |  |  |
| **75** | 9.95 | 11.98 | 18.99 | 10.68 | 14.88 | **0.00** |  |  |  |  |  |  |  |  |  |  |  |  |
| **76** | 16.71 | 19.55 | 17.00 | 16.09 | 16.43 | 15.74 | **0.00** |  |  |  |  |  |  |  |  |  |  |  |
| **77** | 17.49 | 17.58 | 15.11 | 14.63 | 15.41 | 14.64 | 8.57 | **0.00** |  |  |  |  |  |  |  |  |  |  |
| **78** | 18.70 | 18.97 | 16.22 | 15.21 | 16.49 | 16.32 | 9.52 | 6.13 | **0.00** |  |  |  |  |  |  |  |  |  |
| **79** | 18.21 | 20.43 | 8.67 | 16.92 | 15.76 | 16.63 | 15.01 | 13.58 | 13.20 | **0.00** |  |  |  |  |  |  |  |  |
| **80** | 18.45 | 19.04 | 10.67 | 17.76 | 17.55 | 16.31 | 15.96 | 14.46 | 15.28 | 4.61 | **0.00** |  |  |  |  |  |  |  |
| **81** | 25.19 | 22.92 | 11.27 | 20.80 | 20.55 | 21.99 | 18.33 | 16.33 | 17.15 | 11.43 | 11.72 | **0.00** |  |  |  |  |  |  |
| **82** | 20.57 | 22.21 | 10.51 | 17.52 | 17.94 | 18.39 | 16.40 | 14.82 | 14.57 | 8.99 | 9.46 | 10.60 | **0.00** |  |  |  |  |  |
| **83** | 18.24 | 18.77 | 10.49 | 17.61 | 17.18 | 17.16 | 15.04 | 14.51 | 14.14 | 4.43 | 5.41 | 11.46 | 10.09 | **0.00** |  |  |  |  |
| **84** | 20.22 | 22.27 | 5.05 | 17.76 | 17.74 | 18.21 | 14.68 | 15.95 | 16.49 | 9.06 | 8.45 | 12.27 | 10.19 | 9.96 | **0.00** |  |  |  |
| **85** | 19.44 | 20.13 | 6.08 | 18.24 | 16.83 | 17.65 | 15.68 | 14.55 | 16.13 | 10.35 | 11.67 | 12.23 | 11.04 | 11.68 | 7.36 | **0.00** |  |  |
| **86** | 20.41 | 22.42 | 4.40 | 17.61 | 16.20 | 18.13 | 14.34 | 13.41 | 14.01 | 8.80 | 9.87 | 11.75 | 9.32 | 10.34 | 5.90 | 6.03 | **0.00** |  |
| **87** | 23.82 | 25.71 | 20.64 | 22.73 | 23.63 | 23.88 | 20.50 | 19.01 | 18.91 | 20.35 | 19.73 | 22.41 | 20.95 | 22.12 | 20.39 | 20.17 | 22.28 | **0.00** |
